# DPP9 regulates NQO1 and ROS to promote resistance to chemotherapy in liver cancer cells

**DOI:** 10.1016/j.redox.2024.103292

**Published:** 2024-07-29

**Authors:** Yunjiang Zhou, Yaxin Chen, Chenyuan Xuan, Xingyan Li, Yingying Tan, Mengdi Yang, Mengran Cao, Chi Chen, Xing Huang, Rong Hu

**Affiliations:** a State Key Laboratory of Natural Medicines, School of Basic Medicine and Clinical Pharmacy, China Pharmaceutical University, Nanjing, 210009, China; b Department of Pathology, Jiangsu Cancer Hospital & Jiangsu Institute of Cancer Research & Nanjing Medical University Affiliated Cancer Hospital, Nanjing, 210009, China

**Keywords:** DPP9, NQO1, ROS, Liver cancer, Drug resistance

## Abstract

Chemotherapy has been the standard treatment for liver cancer. However, intrinsic or acquired drug resistance remains a major barrier to successful treatment. At present, the underlying molecular mechanisms of chemoresistance in liver cancer have not been elucidated. Dipeptidyl peptidase 9 (DPP9) is a member of the dipeptidyl peptidase IV family that has been found to be highly expressed in a variety of tumors, including liver cancer. It is unclear whether DPP9 affects chemoresistance in liver cancer. In this study, we find that DPP9 weakens the responses of liver cancer cells to chemotherapy drugs by up-regulating NQO1 and inhibiting intracellular ROS levels. In terms of mechanism, DPP9 inhibits ubiquitin-mediated degradation of NRF2 protein by binding to KEAP1, up-regulates NRF2 protein levels, promotes mRNA transcription of NQO1, and inhibits intracellular ROS levels. In addition, the NQO1 inhibitor dicoumarol can enhance the efficacy of chemotherapy drugs in liver cancer cells. Collectively, our findings suggest that inhibiting DPP9/NQO1 signaling can serve as a potential therapeutic strategy for liver cancer.

## 1 Introduction

Liver cancer is one of the most common malignant tumors in the digestive system, primarily consisting of hepatocellular carcinoma, intrahepatic cholangiocarcinoma, and mixed liver cancer. Among them, hepatocellular carcinoma accounts for approximately 90 % of the total. In 2020, there were an estimated 0.906 million new cases (4.7 %) and an estimated 0.83 million deaths (8.3 %) from liver cancer worldwide [1]. At present, chemotherapeutic agents (cisplatin, 5-fluorouracil, doxorubicin, mitomycin, methotrexate, hydroxycamptothecine, etc.) play a critical role in the treatment of liver cancer. However, liver cancer is prone to develop tolerance to chemotherapeutic agents due to the regulation of aberrant signals, which reduces the efficacy of chemotherapeutic agents. Therefore, it is an important issue to study the molecular mechanisms affecting chemoresistance in liver cancer and to find effective therapeutic targets.

Dipeptidyl peptidase 9 (DPP9), a member of the dipeptidyl peptidase IV family, possesses post-proline dipeptidyl aminopeptidase activity, cleaving Xaa-Pro dipeptides from proteins' N-termini [2,3]. DPP9 has a distinctive cellular localization pattern and is widely expressed in cell lines and tissues [4]. Emerging evidence suggests that DPP9 regulates a variety of downstream signals in cells. Huang et al. and Hollingsworth et al. found that DPP9 restrains nucleotide-binding domain and leucine-rich repeat pyrin-domain containing protein 1 (NLRP1) activation by sequestering the C-terminal of NLRP1 [5,6]. Finger et al. revealed that adenylate kinase 2 (AK2) is processed at its N-terminus by DPP9 on its way to the mitochondria, which triggers AK2's rapid proteasomal destruction and prevents the buildup of enzymatically active AK2 in the cytoplasm [7]. Bolgi et al. demonstrated that DPP9 degrades breast cancer-associated protein 2 (BRCA2), encourages the formation of RAD51 foci, and promotes DNA damage repair [8]. Another study showed that DPP9 can interact with FLNA and Syk to create complexes that cause DPP9 to cleave Syk's N-terminal, which has an impact on Syk stability and Syk-dependent signal transduction [9].

During the past few years, studies on the connection between DPP9 and cancer have grown in number. One previous study showed that DPP9 knockdown can prevent the proliferation, migration, and invasion of lung cancer cells, and DPP9 overexpression is a significant independent predictor for poor 5-year overall survival in NSCLC patients [10]. Similar to this, DPP9 overexpression is linked to a worse outcome in colorectal cancer [11]. However, the results of a different investigation on oral squamous cell carcinoma patients revealed the opposite: low DPP9 level was linked to poor prognosis for patients [12]. These findings imply that DPP9 has distinct functions in different types of tumors. DPP9 is abundant in the liver and is up-regulated in the livers of mice with fibrosis and inflammation [13,14]. One previous study showed that decreasing DPP9 or reducing its enzyme activity inhibits Huh7 cell adhesion and migration [15]. Another study showed that DPP9 gene loss-of-function exonic variants are linked to liver cancer [16]. However, it is unclear whether DPP9 may affect chemoresistance in liver cancer. Therefore, in this study, we focused on the effect of DPP9 on chemoresistance in liver cancer and explored related molecular mechanisms.

## 2 Materials and methods

### 2.1 Materials

RPMI 1640, MEM, DMEM, and heat-inactivated FBS were bought from Gibco (Grand Island, NY). β-actin, DPP9, NQO1, and ubiquitin antibodies were bought from Bioworld (Minnesota, USA). Ki67 and Cy3-conjugated goat anti-rabbit IgG antibodies were bought from Servicebio (Wuhan, China). HA, FLAG, NRF2, KEAP1, and HRP-conjugated Goat Anti-Mouse/Rabbit IgG antibodies were bought from Proteintech (Rosemont, IL, USA). ANXA10 and HSPA6 antibodies were bought Abcam (Cambridge, UK). Cycloheximide, MG132, N-acetylcysteine (NAC), dicoumarol, cisplatin, doxorubicin, and 5-FU were bought from Selleck Chemicals (Houston, USA). Protein A + G Agarose, Lipo6000 Transfection Reagent, puromycin, and polybrene were bought from Beyotime (Shanghai, China). KeyFluor 488 Goat Anti-mouse IgG antibody and DAPI were bought from KeyGen (Nanjing, China). All plasmids were constructed by Sangon biotech (Shanghai, China).

### 2.2 Cell lines and cell culture

HCCC-9810, HCCLM3, Hep3B, HepG2, HuH-7, Li-7, SK-Hep-1, SNU-182, SNU-387, SNU-398, and HEK293T cells were obtained from Cell Bank of the Chinese Academy of Sciences (Shanghai, China). HCCC-9810, Li-7, Hep3B, SNU-182, SNU-387, and SNU-398 cells were cultured in RPMI 1640 medium containing 10 % heat-inactivated FBS and 1 % antibiotic (100 mg/mL streptomycin and 100 U/mL penicillin). HCCLM3, HuH-7, SK-Hep-1, and HEK293T cells were cultured in DMEM medium containing 10 % heat-inactivated FBS and 1 % antibiotic (100 mg/mL streptomycin and 100 U/mL penicillin). HepG2 cells were cultured in MEM medium containing 10 % heat-inactivated FBS and 1 % antibiotic (100 mg/mL streptomycin and 100 U/mL penicillin). All the cells were grown at 37 °C in a humidified atmosphere with 5 % CO_2_.

### 2.3 Statistical analysis

Statistical analysis was performed by using SPSS software. Kaplan-Meier analysis was performed for overall survival analyses. Univariate Cox regression and Multivariate Cox regression analysis were performed to analyze the relative risk of patient poor outcome. The results were expressed as mean ± SD. Statistical analysis was performed with using 2-tailed unpaired Student's *t*-test (2 groups), one-way ANOVA, followed by Tukey's test (more than 2 groups), 2-tailed Spearman test, or log-rank test. *P* < 0.05 was considered significant difference.

Detailed methods are provided in Supplemental Methods online.

## 3 Results

### 3.1 DPP9 regulates the responses of liver cancer cells to chemotherapy

**Fig. 1:**
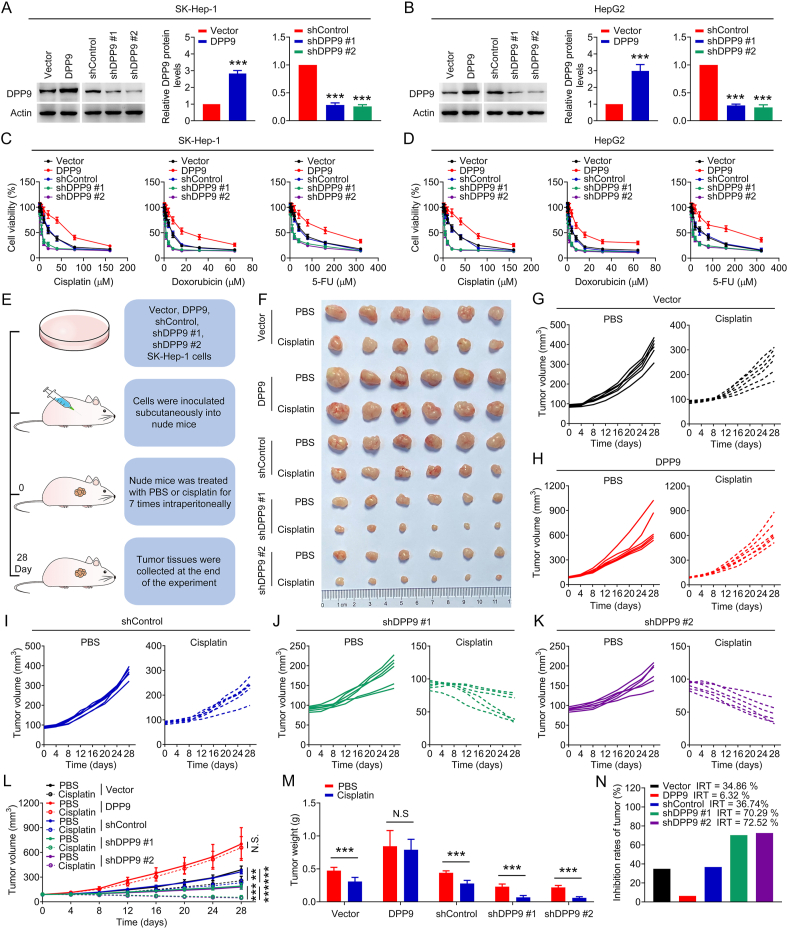
DPP9 regulates the responses of liver cancer cells to chemotherapy. (A–B) DPP9 protein levels in SK-Hep-1 and HepG2 cells transfected with DPP9 overexpression plasmids or DPP9 shRNAs (#1 and #2). (C–D) Toxic effects of chemotherapy drugs (cisplatin, doxorubicin, and 5-FU) on liver cancer cells with DPP9 overexpression or silencing. (E) Schematic diagram of the in vivo study. (F) Image of tumors. (G–L) Growth curves of tumor volume. (M) Tumor weight. (N) Inhibition rates of tumor. Data are shown as mean ± SD. ***P < 0.001 means significant difference *vs.* Vector, shControl or PBS group. N.S. means no significant difference.

To investigate whether DPP9 affects the responses of liver cancer cells to chemotherapy drugs, MTT assay was used to detect the toxic effects of chemotherapy drugs (cisplatin, doxorubicin, and 5-FU) on SK-Hep-1 and HepG2 cells with DPP9 overexpression or silencing (Fig. 1A and B). Fig. 1C and D shows that the efficacy of chemotherapy drugs in cells with DPP9 overexpression was significantly reduced, while the efficacy of chemotherapy drugs in cells with DPP9 silencing was significantly enhanced. Subsequently, we constructed a SK-Hep-1 cell-derived subcutaneous transplanted tumor model in nude mice and observed the growth inhibition effect of cisplatin on xenografts with DPP9 overexpression and silencing (Fig. 1E). As shown in Fig. 1F–N and Supplemental Fig.1, DPP9-overexpressed xenografts showed decreased sensitivity to cisplatin, while DPP9-silenced xenografts showed the opposite. Interestingly, DPP9 overexpression and silencing promoted and inhibited the growth of xenografts, respectively (Fig. 1F–M), indicating that DPP9 can affect the growth of liver cancer. In addition, we detected the DPP9 protein levels in different liver cancer cells and evaluated the efficacy of chemotherapy drugs (cisplatin, doxorubicin, and 5-FU) in cells (Supplemental Fig. 2A-D). Pearson correlation analysis showed that the DPP9 protein level in liver cancer cells was positively correlated with the IC_50_ of chemotherapy drugs (Supplemental Fig. 2E-G). These results indicate that DPP9 increases the resistance of liver cancer cells to chemotherapy drugs.

### 3.2 DPP9 regulates mRNA and protein levels of NQO1 in liver cancer cells

**Fig. 2:**
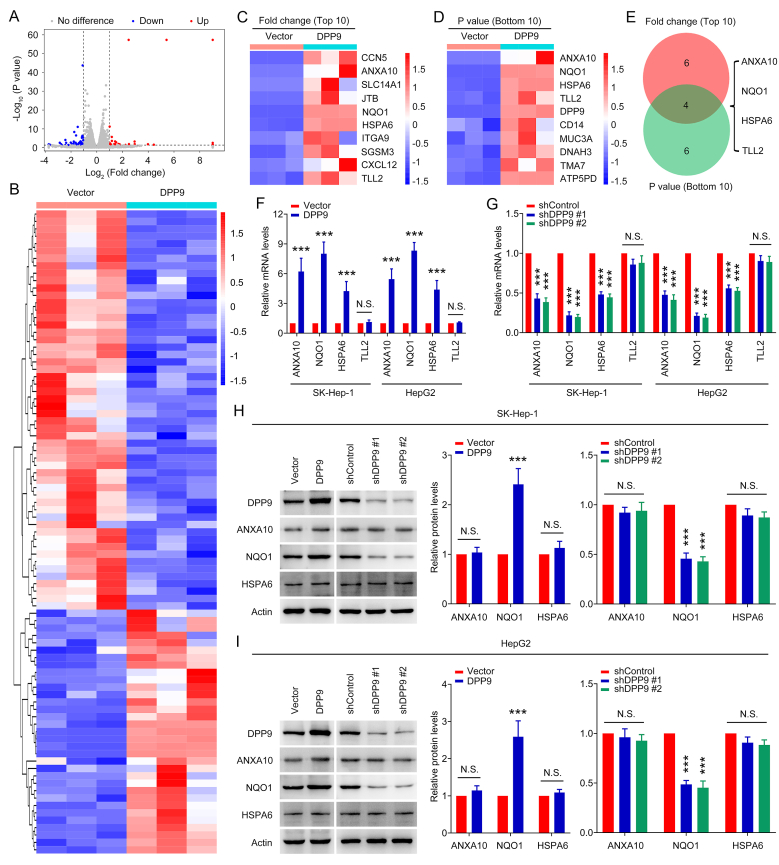
DPP9 regulates mRNA and protein levels of NQO1 in liver cancer cells. (A) Volcano plots of different gene expression in SK-Hep-1 cells with or without DPP9 overexpression. (B) Heatmap of 54 down-regulated genes and 33 up-regulated genes in SK-Hep-1 cells with or without DPP9 overexpression. (C) Heatmap of 10 genes with the highest up-regulation fold change. (D) Heatmap of 10 genes with the lowest P-value. (E) Venn gram of intersection of 10 genes with the highest up-regulation fold change and 10 genes with the lowest P-value. (F) ANXA10, NQO1, HSPA6, and TLL2 mRNA levels in SK-Hep-1 and HepG2 cells with or without DPP9 overexpression. (G) ANXA10, NQO1, HSPA6, and TLL2 mRNA levels in SK-Hep-1 and HepG2 cells with or without DPP9 silencing. (H) ANXA10, NQO1, and HSPA6 protein levels in SK-Hep-1 cells with DPP9 overexpression or silencing. (I) ANXA10, NQO1, and HSPA6 protein levels in HepG2 cells with DPP9 overexpression or silencing. Data are shown as mean ± SD. ***P < 0.001 means significant difference *vs.* Vector or shControl group. N.S. means no significant difference.

To investigate which genes in liver cancer cells are regulated by DPP9, we performed RNA sequencing on SK-Hep-1 cells with and without DPP9 overexpression. As shown in Fig. 2A and B, compared with normal SK-Hep-1 cells, 54 genes in SK-Hep-1 cells with DPP9 overexpression were significantly down-regulated and 33 genes were markedly up-regulated. We further performed GO and KEGG enrichment analyses for these genes, and the results showed that these genes were enriched in pathways involving signal transduction, cell adhesion, transcriptional misregulation in cancer, etc. (Supplemental Fig. 3). Among 33 up-regulated genes, four genes (ANXA10, NQO1, HSPA6, and TLL2) were obtained by crossing 10 genes with the highest up-regulation fold change and the 10 genes with the lowest P-value (Fig. 2C–E). Subsequently, we used the qPCR assay to measure mRNA levels of ANXA10, NQO1, HSPA6, and TLL2 in SK-Hep-1 and HepG2 cells with DPP9 overexpression or silencing. Fig. 2F and G shows that mRNA levels of ANXA10, NQO1, and HSPA6 in cells with DPP9 overexpression and silencing were significantly up-regulated and down-regulated, respectively, while mRNA levels of TLL2 remained unchanged. Next, Western blot assay was used to detect protein levels of ANXA10, NQO1, and HSPA6 in cells with DPP9 overexpression or silencing. As shown in Fig. 2H and I, NQO1 protein levels in cells with DPP9 overexpression and silencing were significantly up-regulated and down-regulated, respectively, while protein levels of ANXA10 and HSPA6 were not significantly changed. These results suggest that DPP9 can regulate the mRNA and protein levels of NQO1 in liver cancer cells.

### 3.3 DPP9 promotes resistance to chemotherapy in liver cancer cells by regulating NQO1 and ROS levels

**Fig. 3:**
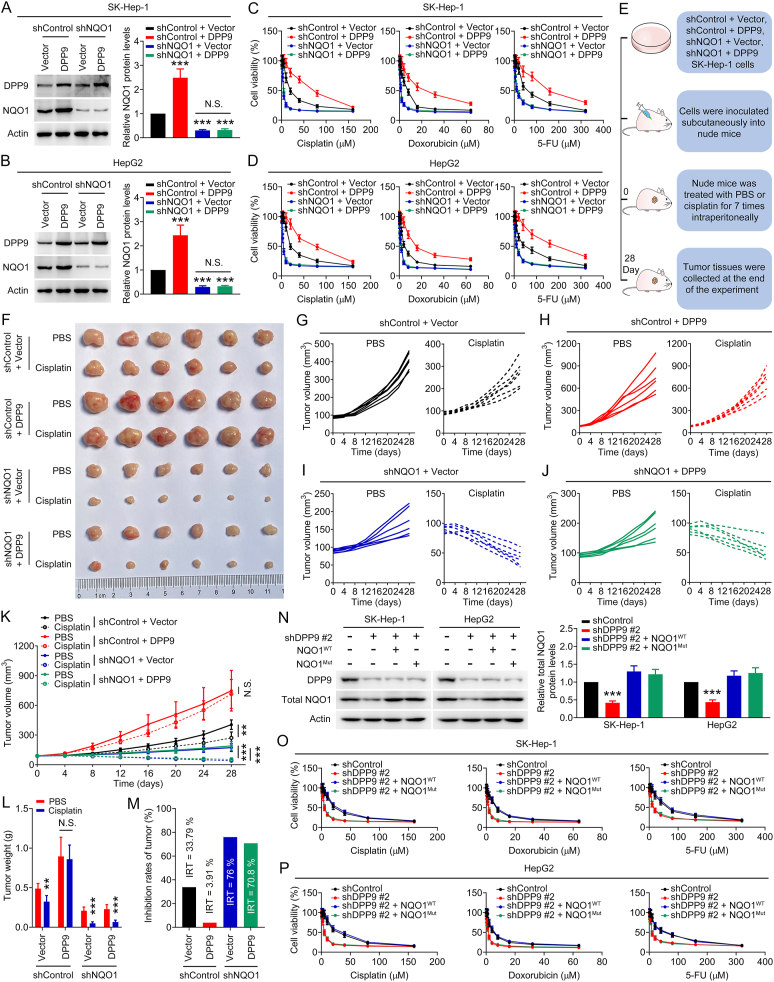
DPP9-mediated chemoresistance in liver cancer cells is NQO1-dependent. (A–B) NQO1 protein levels in SK-Hep-1 and HepG2 cells transfected with shControl + Vector, shControl + DPP9, shNQO1 + Vector, and shNQO1 + DPP9. (C–D) Toxic effects of chemotherapy drugs (cisplatin, doxorubicin, and 5-FU) on liver cancer cells transfected with shControl + Vector, shControl + DPP9, shNQO1 + Vector, and shNQO1 + DPP9. (E) Schematic diagram of the in vivo study. (F) Image of tumors. (G–K) Growth curves of tumor volume. (L) Tumor weight. (M) Inhibition rates of tumor. (N) NQO1^WT^ and NQO1^Mut^ compensated for total NQO1 protein levels in SK-Hep-1 and HepG2 cells with DPP9 knockdown. (O–P) Toxic effects of chemotherapy drugs (cisplatin, doxorubicin, and 5-FU) on SK-Hep-1 and HepG2 cells transfected with shControl, shDPP9 #2, shDPP9 #2 + NQO1^WT^, and shDPP9 #2 + NQO1^Mut^. Data are shown as mean ± SD. **P < 0.01 or ***P < 0.001 means significant difference *vs.* shControl + Vector, PBS, or shControl group. N.S. means no significant difference.

**Fig. 4:**
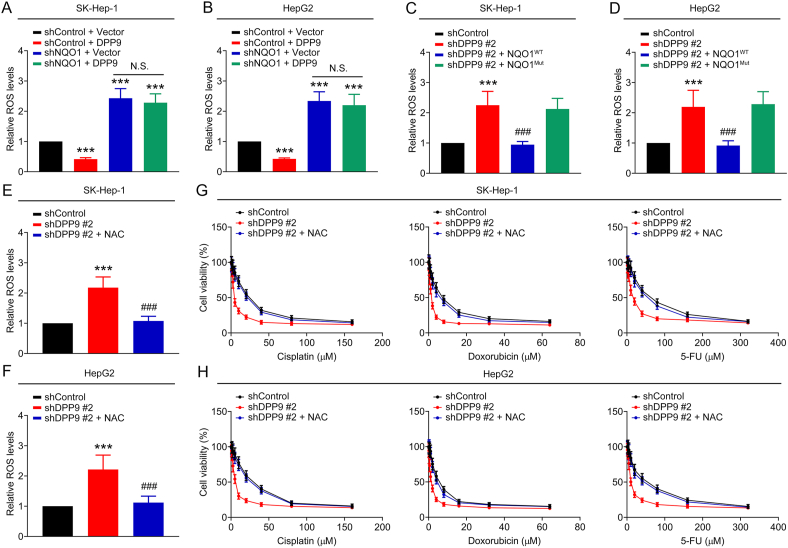
DPP9 promotes chemoresistance by inhibiting ROS levels in liver cancer cells. (A–B) The ROS levels in SK-Hep-1 and HepG2 cells transfected with shControl + Vector, shControl + DPP9, shNQO1 + Vector, and shNQO1 + DPP9. (C–D) The ROS levels in SK-Hep-1 and HepG2 cells transfected with shControl, shDPP9 #2, shDPP9 #2 + NQO1^WT^, and shDPP9 #2 + NQO1^Mut^. (E–F) The ROS levels in SK-Hep-1 and HepG2 cells of the shControl, shDPP9 #2, and shDPP9 #2 + NAC (5 mM) groups. (G–H) Toxic effects of chemotherapy drugs (cisplatin, doxorubicin, and 5-FU) on liver cancer cells of the shControl, shDPP9 #2, and shDPP9 #2 + NAC (5 mM) groups. Data are shown as mean ± SD. ***P < 0.001 means significant difference *vs.* shControl + Vector or shControl group. ^###^*P <* 0.001 means significant difference *vs.* shDPP9 #2 group. N.S. means no significant difference.

Previous studies have shown that up-regulation of NQO1 can increase chemoresistance in cancer cells [[17], [18], [19], [20], [21]]. Since DPP9 can regulate NQO1 in liver cancer cells, does DPP9 affect chemoresistance in liver cancer cells by regulating NQO1? To answer this question, we overexpressed DPP9 in SK-Hep-1 and HepG2 cells with or without NQO1 knockdown (Fig. 3A and B) and used the MTT assay to detect cell responses to chemotherapy drugs. As shown in Fig. 3C and D, after DPP9 in SK-Hep-1 and HepG2 cells without NQO1 knockdown was overexpressed, the toxic effect of chemotherapy drugs on cells would be significantly weakened. However, overexpression of DPP9 did not significantly alter the responses of NQO1-silenced cells to chemotherapy drugs. Next, we constructed a SK-Hep-1 cell-derived subcutaneous transplanted tumor model in nude mice for in vivo verification (Fig. 3E). As shown in Fig. 3F–M and Supplemental Fig. 4, DPP9 overexpression significantly reduced the sensitivity of xenografts without NQO1 knockdown to cisplatin, but did not affect the efficacy of cisplatin in NQO1-silenced xenografts. Interestingly, Fig. 3F–K shows that NQO1 silencing could inhibit the growth of xenografts, indicating that NQO1 can promote the growth of liver cancer. In addition, we supplemented NQO1^WT^ and NQO1^Mut^ (Enzyme functional inactivation) in SK-Hep-1 and HepG2 cells with DPP9 knockdown and evaluated the cytotoxicity of chemotherapy drugs using MTT assay. As shown in Fig. 3N–P, supplementing NQO1^WT^ could reduce DPP9 knockdown-mediated chemotherapy sensitization, while supplementation with NQO1^Mut^ did not have a similar effect. Interestingly, we found that DPP9 could regulate ROS levels in liver cancer cells, and this regulatory effect was dependent on NQO1 (Fig. 4A–D). Moreover, ROS scavenger N-acetylcysteine (NAC) could reduce DPP9 knockdown-mediated chemotherapy sensitization (Fig. 4E–H). These results suggest that DPP9 promotes chemoresistance in liver cancer cells by up-regulating NQO1 and inhibiting ROS levels.

### 3.4 DPP9 and NQO1 are highly expressed in liver cancer and are closely associated with poor prognosis

**Fig. 5:**
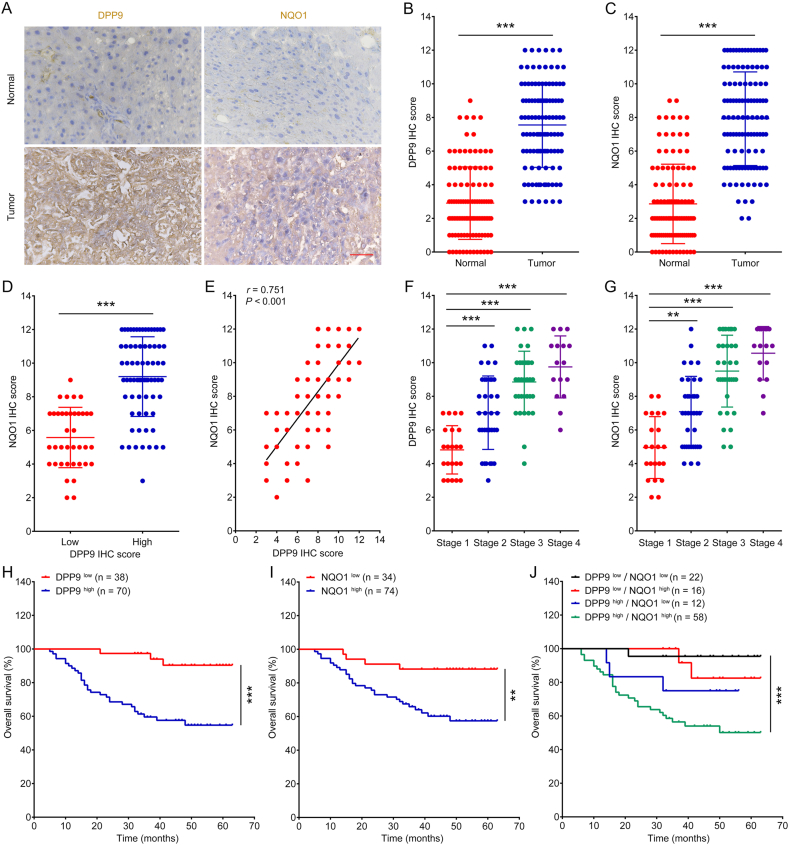
DPP9 and NQO1 are highly expressed in liver cancer and are closely associated with poor prognosis. (A–C) DPP9 and NQO1 IHC scores in tumor tissues and adjacent normal tissues of liver cancer. Scale bar = 50 μm. (D) NQO1 IHC scores in tumor tissues with low or high DPP9 IHC score. (E) Spearman correlation analysis of DPP9 and NQO1 IHC scores in tumor tissues. (F and G) DPP9 and NQO1 IHC scores in tumor tissues with clinical stage 1, stage 2, stage 3, and stage 4. (H–J) Kaplan-Meier survival analysis. Data are shown as mean ± SD. **P < 0.01 or ***P < 0.001 means significant difference.

DPP9 and NQO1 protein levels in liver cancer and adjacent normal tissues were detected by using immunohistochemical (IHC) assay. Fig. 5A–C illustrate that liver cancer tumor tissues had considerably greater DPP9 and NQO1 IHC scores than the adjacent normal tissues. Moreover, tumor tissues with a high DPP9 score had a significantly higher NQO1 IHC score than tumor tissues with a low DPP9 score (Fig. 5D). Spearman correlation analysis revealed that the IHC scores of these two proteins were substantially associated in liver cancer tissues (Fig. 5E). In addition, we analyzed the IHC scores of DPP9 and NQO1 in tumor tissues at different clinical stages. Fig. 5F and G shows that tumor tissues from stages 2–4 had considerably higher DPP9 and NQO1 IHC scores than those from stage 1 tumor tissues. Fig. 5H and I shows that liver cancer patients with a high DPP9 IHC score or a high NQO1 IHC score in tumor tissue had a poor survival prognosis. Next, we divided patients into four groups: DPP9 ^low^/NQO1^low^, DPP9^low^/NQO1^high^, DPP9^high^/NQO1^low^, and DPP9^high^/NQO1^high^, and analyzed the survival of patients in each group. As shown in Fig. 5J, patients in the DPP9^high^/NQO1^high^ group had the shortest overall survival. According to the results of the univariate and multivariate Cox regression analyses, patients with DPP9^high^/NQO1^high^ IHC scores had a markedly high risk (Supplemental Table 1). These results suggest that DPP9 and NQO1 are highly expressed in liver cancer and are closely associated with poor prognosis.

### 3.5 DPP9 promotes the expression of NQO1 in liver cancer cells by regulating NRF2

**Fig. 6:**
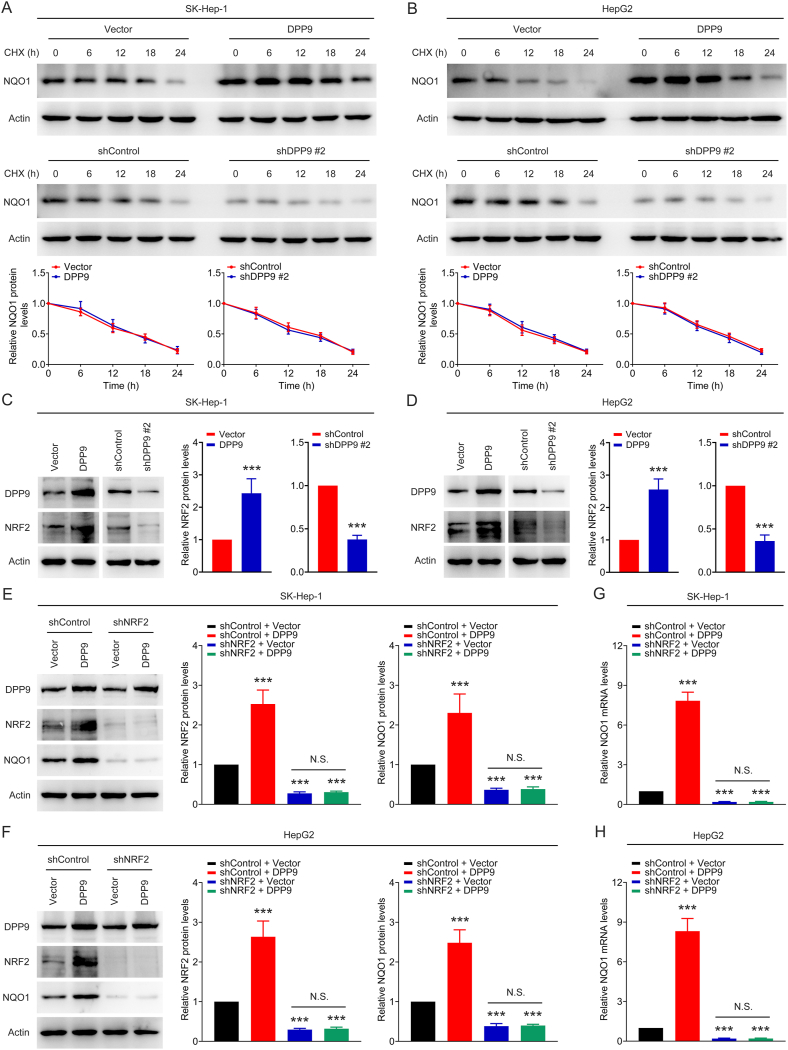
DPP9 promotes the expression of NQO1 in liver cancer cells by regulating NRF2. (A–B) NQO1 protein degradation rate in SK-Hep-1 and HepG2 cells with DPP9 overexpression and silencing. (C–D) NRF2 protein levels in SK-Hep-1 and HepG2 cells with DPP9 overexpression and silencing. (E–F) NRF2 and NQO1 protein levels in SK-Hep-1 and HepG2 cells transfected with shControl + Vector, shControl + DPP9, shNRF2 + Vector, and shNRF2 + DPP9. (G–H) NQO1 mRNA levels in SK-Hep-1 and HepG2 cells transfected with shControl + Vector, shControl + DPP9, shNRF2 + Vector, and shNRF2 + DPP9. Data are shown as mean ± SD. **P < 0.01 or ***P < 0.001 means significant difference *vs.* Vector, shControl, or shControl + Vector group. N.S. means no significant difference.

Since we have shown that DPP9 can regulate the mRNA levels of NQO1 in liver cancer cells, the question arises as to whether DPP9 also has an impact on the protein stability of NQO1. As shown in Fig. 6A and B, there was no discernible difference in the rate of NQO1 protein degradation in SK-Hep-1 and HepG2 cells following the overexpression and silencing of DPP9, indicating that DPP9 does not affect the levels of NQO1 protein in liver cancer cells by regulating the stability of NQO1 protein. It has been shown that NRF2 is a critical upstream transcription factor of NQO1 [[22], [23], [24]]. We then asked if DPP9 regulates the expression of NQO1 through NRF2. Firstly, NRF2 protein levels in SK-Hep-1 and HepG2 cells with DPP9 overexpression and silencing were detected by using Western blot assay. Fig. 6C and D shows that overexpression of DPP9 significantly up-regulated NRF2 protein levels in cells, and silencing of DPP9 significantly down-regulated NRF2 protein levels, indicating that DPP9 can regulate NRF2 protein levels in liver cancer cells. Next, DPP9 overexpression plasmids were used to overexpress DPP9 in NRF2-silenced cells. Western blot and qPCR experiments were performed to detect NQO1 protein and mRNA levels in cells. As shown in Fig. 6E–H, DPP9 significantly up-regulated NQO1 protein and mRNA levels in NRF2-unsilenced cells. However, there was no significant regulatory effect on NQO1 protein and mRNA levels in NRF2-silenced cells. These results suggest that the regulation of NQO1 in liver cancer cells by DPP9 depends on NRF2.

### 3.6 DPP9 inhibits NRF2 ubiquitination degradation by binding KEAP1 in liver cancer cells

Recently, Chang et al. found that DPP9 inhibited the ubiquitination degradation of NRF2 by binding KEAP1 in Clear Cell Renal Cell Carcinoma, thereby regulating the protein level of NRF2 [25]. So, how does DPP9 regulate NRF2 protein levels in liver cancer cells? The NRF2 mRNA levels in SK-Hep-1 and HepG2 cells that overexpressed and silenced DPP9 were detected by using qPCR assay. As shown in Supplemental Fig. 5A and B, when DPP9 was overexpressed or silenced in cells, the mRNA levels of NRF2 did not change significantly, indicating that DPP9 does not affect mRNA level of NRF2. Next, we investigated the effect of DPP9 on NRF2 degradation in liver cancer cells and explored the related molecular mechanisms. The results in Supplemental Fig. 5-7 show that DPP9 bound to KEAP1 through its ESGE motif in liver cancer cells, inhibited NRF2 ubiquitination degradation and up-regulated NRF2 protein levels. These results are basically consistent with those of Chang et al.

### 3.7 Dicoumarol enhances the efficacy of chemotherapy drugs in liver cancer cells

**Fig. 7:**
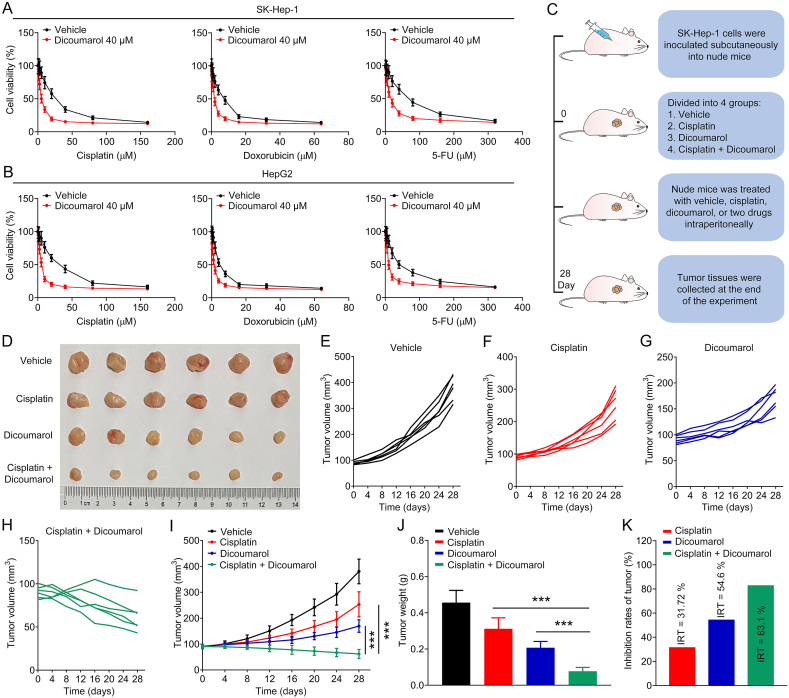
Dicoumarol enhances the efficacy of chemotherapy drugs in liver cancer cells. (A–B) Toxic effects of chemotherapy drugs (cisplatin, doxorubicin, and 5-FU) on SK-Hep-1 and HepG2 cells treated with or without 40 μM of dicoumarol. (C) Schematic diagram of the in vivo study. (D) Image of tumors. (E–I) Growth curves of tumor volume. (J) Tumor weight. (K) Inhibition rates of tumor. Data are shown as mean ± SD. ***P < 0.001 means significant difference.

Next, we evaluated the effect of dicoumarol (an NQO1 inhibitor) on the efficacy of chemotherapy drugs in liver cancer cells. As shown in Fig. 7A–B, dicoumarol could increase the sensitivity of liver cancer cells to chemotherapy drugs (cisplatin, doxorubicin, and 5-FU). Subsequently, we constructed a SK-Hep-1 cell-derived subcutaneous transplanted tumor model in nude mice and observed the growth inhibition effect of dicoumarol combined with cisplatin on xenografts (Fig. 7C). As shown in Fig. 7D–K, the growth inhibitory effect of these combination was significantly stronger than that of either drug alone. These results suggest that dicoumarol can increase the efficacy of chemotherapy drugs in liver cancer cells.

## 4 Discussion

**Fig. 8:**
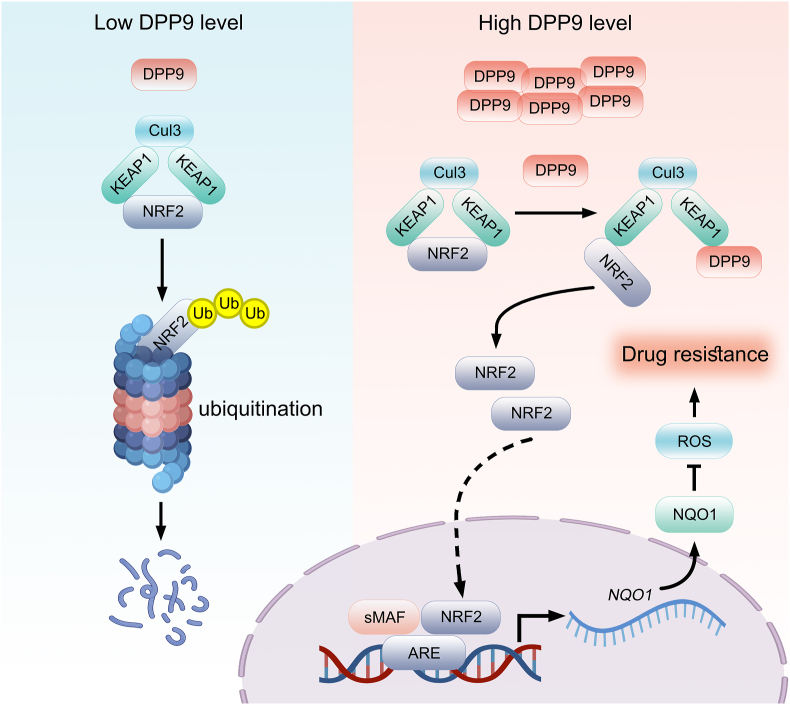
Schematic diagram of DPP9-mediated chemoresistance in liver cancer cells. DPP9 inhibits ubiquitin-mediated degradation of NRF2 protein by binding to KEAP1, up-regulates NRF2 protein levels, promotes mRNA transcription of NQO1, inhibits intracellular ROS levels, and thus weakens the responses of liver cancer to chemotherapy drugs.

Excessive activation of aberrant signals can weaken the response of liver cancer to chemotherapy drugs, resulting in reduced drug efficacy. Despite extensive research on the causes of drug resistance in liver cancer, the precise molecular mechanism remains unclear. This study reveals that DPP9 inhibits ubiquitin-mediated degradation of NRF2 protein by binding to KEAP1, up-regulates NRF2 protein levels, promotes mRNA transcription of NQO1, inhibits intracellular ROS levels, and thus weakens the responses of liver cancer to chemotherapy drugs (Fig. 8). Interestingly, Chang et al. have previously reported similar results [25]. Unlike our findings, they found that DPP9 inhibited ferroptosis and induced sorafenib resistance in Clear Cell Renal Cell Carcinoma not by regulating the NRF2 downstream target gene NQO1, but by regulating SLC7A11. We speculated that this difference might be closely related to the specificity of the tumor. Although somewhat different, both our study and Chang et al. demonstrate that DPP9 can potentially serve as a therapeutic target for treating cancer.

NQO1, a phase II detoxification enzyme regulated by the transcription factor NRF2, protects cells from xenobiotics and oxidative damage by catalyzing the reduction of quinone substrates. It has been shown that NQO1 is overexpressed in many human tumor tissues [[26], [27], [28], [29], [30]] and its overexpression leads to resistance to chemotherapy in a variety of tumors [[17], [18], [19], [20], [21]]. Distinct mechanisms are involved in NQO1-mediated chemoresistance in cancer. NQO1 has been reported to be resistant to 5-FU, doxorubicin, and gemcitabine in cholangiocarcinoma by modulating p53 [20]. In addition, as a scavenger of superoxide anion radicals, NQO1 triggers drug resistance in cancer cells by mitigating intracellular ROS [17]. Beyond what has been mentioned above, it has also been demonstrated that NQO1 is involved in drug metabolism, ultimately leading to drug resistance [31,32]. In this study, we find that DPP9 can inhibit ROS levels in liver cancer cells by up-regulating NQO1. However, it is not clear how up-regulated NQO1 regulates ROS levels in liver cancer cells. Since the ratio of intracellular NAD(P)H/NAD(P)^+^ plays an important role in regulating ROS levels to maintain redox homeostasis [33], up-regulation of NQO1 may inhibit ROS levels by influencing the ratio in liver cancer cells. Previous studies have shown that inhibiting ROS can reduce oxidative DNA damage and increase DNA damage repair [34,35]. Therefore, we speculated that higher DPP9 or NQO1 might alleviate oxidative DNA damage and increase DNA damage repair by inhibiting ROS to promote resistance to chemotherapy. Considering the important role of intracellular ROS levels in DPP9 and NQO1-mediated chemoresistance, DPP9 and NQO1 might not induce resistance to drugs that do not increase ROS levels in cells. It is worth noting that NQO1 polymorphism is also a consideration for drug resistance [36]. However, the mechanism by which NQO1 polymorphism promotes drug resistance is different. Primarily, NQO1 polymorphism weakens the activity of its enzyme, resulting in NQO1-bioactivating drugs (such as β-lapachone) not being activated and not being effective.

Previous studies have shown that NRF2 can regulate numerous downstream target genes related to antioxidant activity, glutathione synthesis and conjugation, drug-metabolizing enzymes, and transporters in cells [37,38]. Since DPP9 significantly increases NRF2 protein levels in liver cancer cells, it can theoretically up-regulate the transcription of these downstream target genes as well. However, RNA sequencing result shows that apart from NQO1 gene, there is no significant up-regulation observed for other NRF2 downstream target genes in liver cancer cells with DPP9 overexpression. shKEAP1 or NRF2 agonist CDDO-me was then used to mimic DPP9 overexpression to see whether only NQO1 could be up-regulated in cells. We found that KEAP1 knockdown could significantly increase the levels of NRF2 downstream target genes (ABCC1, ABCC2, AKR1C1, AKR1C3, CAT, GCLC, GCLM, GSTM2, HO-1, NQO1, and SOD2), while CDDO-me could up-regulate the levels of other NRF2 downstream target genes except for GSTM2 and SOD2 in cells (Supplemental Fig. 8 and 9). We speculated that the reason why CDDO-me had no effect on mRNA levels of GSTM2 and SOD2 in cells was that CDDO-me might balance intracellular GSTM2 and SOD2 mRNA levels by acting on other targets, resulting in unchanged mRNA levels of these two genes. Similar to CDDO-me, DPP9 might also balance the mRNA levels of NRF2 downstream target genes by acting on other intracellular targets, resulting in no significant changes of other target genes except for NQO1.

It has been shown that KEAP1 combines with NRF2 in cells to form two conformations: closed and open states [39,40]. In the closed state, both NRF2's DLG and ETGE motifs are attached to a KEAP1 dimer, while in the open state, only NRF2's ETGE motif binds to a single KEAP1 molecule. The conversion of the KEAP1-NRF2 complex conformation from “open” to “closed” triggers ubiquitin-mediated degradation of NRF2 protein. DPP9 contains ETGE and ESGE motifs. Theoretically, both motifs of DPP9 may bind KEAP1 and interfere with the binding of KEAP1 and NRF2. However, both our study and Chang et al. [25] demonstrate that DPP9 interacts with KEAP1 through its ESGE motif rather than its ETGE motif. Since DPP9 inhibits the binding of KEAP1 and NRF2, it is not surprising that DPP9 exhibits a significant promoting effect on NRF2 stabilization. In addition to DPP9, other proteins can also regulate NRF2 stability by competing with NRF2 for binding KEAP1. For example, PALB2 [41], CDK20 [42], FAM117B [43], and WTX [44] can regulate the stability of NRF2 by combining their ETGE motifs with KEAP1. Nestin can regulate the stability of NRF2 by combining its ESGE and DLG motifs with KEAP1 [45]. IASPP and aPKCι can directly bind to KEAP1 and regulate NRF2 stability via DLT and DLL motifs, respectively [46,47]. SQSTM1 binds to KEAP1 through its STGE motif and regulates the stability of NRF2 [48]. These studies suggest that proteins containing ETGE, DLG or similar sequences have the potential to bind KEAP1 and regulate the stability of NRF2 protein.

Based on this research, the combination of small-molecule inhibitors that disrupt the DPP9-KEAP1 complex with chemotherapy drugs could potentially improve the response of liver cancer patients to chemotherapy and prolong their survival. Given DPP9's critical function in liver cancer, designing small-molecule inhibitors may offer a fresh method of treating this disease.

## CRediT authorship contribution statement

**Yunjiang Zhou:** Writing – original draft, Visualization, Validation, Investigation, Funding acquisition, Formal analysis, Data curation. **Yaxin Chen:** Visualization, Validation, Methodology, Investigation, Data curation. **Chenyuan Xuan:** Investigation. **Xingyan Li:** Investigation. **Yingying Tan:** Investigation. **Mengdi Yang:** Investigation. **Mengran Cao:** Investigation. **Chi Chen:** Investigation. **Xing Huang:** Validation, Supervision, Methodology, Conceptualization. **Rong Hu:** Writing – review & editing, Supervision, Resources, Project administration, Funding acquisition, Conceptualization.

## Declaration of competing interest

The authors declare no potential conflicts of interest.

## Data Availability

Data will be made available on request.

## References

[1] Sung H., Ferlay J., Siegel R.L., Laversanne M., Soerjomataram I., Jemal A., Bray F. (2021). Global cancer statistics 2020: GLOBOCAN estimates of incidence and mortality worldwide for 36 cancers in 185 countries. CA A Cancer J. Clin..

[2] Geiss-Friedlander R., Parmentier N., Möller U., Urlaub H., Van den Eynde B.J., Melchior F. (2009). The cytoplasmic peptidase DPP9 is rate-limiting for degradation of proline-containing peptides,. J. Biol. Chem..

[3] Zhang H., Maqsudi S., Rainczuk A., Duffield N., Lawrence J., Keane F.M., Justa-Schuch D., Geiss-Friedlander R., Gorrell M.D., Stephens A.N. (2015). Identification of novel dipeptidyl peptidase 9 substrates by two-dimensional differential in-gel electrophoresis. FEBS J..

[4] Zhang H., Chen Y., Keane F.M., Gorrell M.D. (2013). Advances in understanding the expression and function of dipeptidyl peptidase 8 and 9. Mol. Cancer Res..

[5] Huang M., Zhang X., Toh G.A., Gong Q., Wang J., Han Z., Wu B., Zhong F., Chai J. (2021). Structural and biochemical mechanisms of NLRP1 inhibition by DPP9. Nature.

[6] Hollingsworth L.R., Sharif H., Griswold A.R., Fontana P., Mintseris J., Dagbay K.B., Paulo J.A., Gygi S.P., Bachovchin D.A., Wu H. (2021). DPP9 sequesters the C terminus of NLRP1 to repress inflammasome activation. Nature.

[7] Finger Y., Habich M., Gerlich S., Urbanczyk S., van de Logt E., Koch J., Schu L., Lapacz K.J., Ali M., Petrungaro C., Salscheider S.L., Pichlo C., Baumann U., Mielenz D., Dengjel J., Brachvogel B., Hofmann K., Riemer J. (2020). Proteasomal degradation induced by DPP9-mediated processing competes with mitochondrial protein import. EMBO J..

[8] Bolgi O., Silva-Garcia M., Ross B., Pilla E., Kari V., Killisch M., Spitzner M., Stark N., Lenz C., Weiss K., Donzelli L., Gorrell M.D., Grade M., Riemer J., Urlaub H., Dobbelstein M., Huber R., Geiss-Friedlander R. (2022). Dipeptidyl peptidase 9 triggers BRCA2 degradation and promotes DNA damage repair. EMBO Rep..

[9] Justa-Schuch D., Silva-Garcia M., Pilla E., Engelke M., Kilisch M., Lenz C., Möller U., Nakamura F., Urlaub H., Geiss-Friedlander R. (2016). DPP9 is a novel component of the N-end rule pathway targeting the tyrosine kinase Syk. Elife.

[10] Tang Z., Li J., Shen Q., Feng J., Liu H., Wang W., Xu L., Shi G., Ye X., Ge M., Zhou X., Ni S. (2017). Contribution of upregulated dipeptidyl peptidase 9 (DPP9) in promoting tumoregenicity, metastasis and the prediction of poor prognosis in non-small cell lung cancer (NSCLC). Int. J. Cancer.

[11] Saso K., Miyoshi N., Fujino S., Sasaki M., Yasui M., Ohue M., Ogino T., Takahashi H., Uemura M., Matsuda C., Mizushima T., Doki Y., Eguchi H. (2020). Dipeptidyl peptidase 9 increases chemoresistance and is an indicator of poor prognosis in colorectal cancer. Ann. Surg Oncol..

[12] Wu Q.Q., Zhao M., Huang G.Z., Zheng Z.N., Chen Y., Zeng W.S., Lv X.Z. (2020). Fibroblast activation protein (FAP) overexpression induces epithelial-mesenchymal transition (EMT) in oral squamous cell carcinoma by down-regulating dipeptidyl peptidase 9 (DPP9). OncoTargets Ther..

[13] Yu D.M., Ajami K., Gall M.G., Park J., Lee C.S., Evans K.A., McLaughlin E.A., Pitman M.R., Abbott C.A., McCaughan G.W., Gorrell M.D. (2009). The in vivo expression of dipeptidyl peptidases 8 and 9,. J. Histochem. Cytochem..

[14] Chowdhury S., Chen Y., Yao T.W., Ajami K., Wang X.M., Popov Y., Schuppan D., Bertolino P., McCaughan G.W., Yu D.M., Gorrell M.D. (2013). Regulation of dipeptidyl peptidase 8 and 9 expression in activated lymphocytes and injured liver. World J. Gastroenterol..

[15] Zhang H., Chen Y., Wadham C., McCaughan G.W., Keane F.M., Gorrell M.D. (2015). Dipeptidyl peptidase 9 subcellular localization and a role in cell adhesion involving focal adhesion kinase and paxillin. Biochim. Biophys. Acta.

[16] Huang J.C., Emran A.A., Endaya J.M., McCaughan G.W., Gorrell M.D., Zhang H.E. (2021).

[17] Xue W., Wang T., Tian W.J., Pang S.Q., Zhang H.F., Jia W.D. (2024). NQO1 mediates lenvatinib resistance by regulating ROS-induced apoptosis in hepatocellular carcinoma. Curr Med Sci.

[18] Madajewski B., Boatman M.A., Martinez I., Carter J.H., Bey E.A. (2023). NAD(P)H quinone oxidoreductase-1 expression promotes self-renewal and therapeutic resistance in non-small cell lung cancer. Genes.

[19] Zhang K., Chen D., Ma K., Wu X., Hao H., Jiang S. (2018). NAD(P)H: quinone oxidoreductase 1 (NQO1) as a therapeutic and diagnostic target in cancer. J. Med. Chem..

[20] Zeekpudsa P., Kukongviriyapan V., Senggunprai L., Sripa B., Prawan A. (2014). Suppression of NAD(P)H-quinone oxidoreductase 1 enhanced the susceptibility of cholangiocarcinoma cells to chemotherapeutic agents. J. Exp. Clin. Cancer Res..

[21] Buranrat B., Prawan A., Kukongviriyapan U., Kongpetch S., Kukongviriyapan V. (2010). Dicoumarol enhances gemcitabine-induced cytotoxicity in high NQO1-expressing cholangiocarcinoma cells. World J. Gastroenterol..

[22] Venugopal R., Jaiswal A.K. (1996). Nrf1 and Nrf2 positively and c-Fos and Fra1 negatively regulate the human antioxidant response element-mediated expression of NAD(P)H:quinone oxidoreductase1 gene. Proc. Natl. Acad. Sci. U. S. A..

[23] Dhakshinamoorthy S., Jaiswal A.K. (2000). Small maf (MafG and MafK) proteins negatively regulate antioxidant response element-mediated expression and antioxidant induction of the NAD(P)H: quinone oxidoreductase1 gene. J. Biol. Chem..

[24] Bloom D., Dhakshinamoorthy S., Jaiswal A.K. (2002). Site-directed mutagenesis of cysteine to serine in the DNA binding region of Nrf2 decreases its capacity to upregulate antioxidant response element-mediated expression and antioxidant induction of NAD(P)H:quinone oxidoreductase1 gene. Oncogene.

[25] Chang K., Chen Y., Zhang X., Zhang W., Xu N., Zeng B., Wang Y., Feng T., Dai B., Xu F., Ye D., Wang C. (2023). DPP9 stabilizes NRF2 to suppress ferroptosis and induce sorafenib resistance in clear cell renal cell carcinoma. Cancer Res..

[26] Yang Y., Zhang Y., Wu Q., Cui X., Lin Z., Liu S., Chen L. (2014). Clinical implications of high NQO1 expression in breast cancers. J. Exp. Clin. Cancer Res..

[27] Oh E.T., Kim J.W., Kim J.M., Kim S.J., Lee J.S., Hong S.S., Goodwin J., Ruthenborg R.J., Jung M.G., Lee H.J., Lee C.H., Park E.S., Kim C., Park H.J. (2016). NQO1 inhibits proteasome-mediated degradation of HIF-1α. Nat. Commun..

[28] Siegel D., Franklin W.A., Ross D. (1998). Immunohistochemical detection of NAD(P)H:quinone oxidoreductase in human lung and lung tumors. Clin. Cancer Res..

[29] Lin L., Qin Y., Jin T., Liu S., Zhang S., Shen X., Lin Z. (2014). Significance of NQO1 overexpression for prognostic evaluation of gastric adenocarcinoma. Exp. Mol. Pathol..

[30] Wang X., Liu Y., Han A., Tang C., Xu R., Feng L., Yang Y., Chen L., Lin Z. (2022). The NQO1/p53/SREBP1 axis promotes hepatocellular carcinoma progression and metastasis by regulating Snail stability,. Oncogene.

[31] Verma H., Singh Bahia M., Choudhary S., Kumar Singh P., Silakari O. (2019). Drug metabolizing enzymes-associated chemo resistance and strategies to overcome it. Drug Metab. Rev..

[32] Raju B., Choudhary S., Narendra G., Verma H., Silakari O. (2021). Molecular modeling approaches to address drug-metabolizing enzymes (DMEs) mediated chemoresistance: a review. Drug Metab. Rev..

[33] Shen A., Kim H.J., Oh G.S., Lee S.B., Lee S., Pandit A., Khadka D., Sharma S., Kim S.Y., Choe S.K., Yang S.H., Cho E.Y., Shim H., Park R., Kwak T.H., So H.S. (2018). Pharmacological stimulation of NQO1 decreases NADPH levels and ameliorates acute pancreatitis in mice. Cell Death Dis..

[34] Burke R., Chu C., Zhou G.D., Putluri V., Putluri N., Stading R.E., Couroucli X., Lingappan K., Moorthy B. (2021). Role of human NADPH quinone oxidoreductase (NQO1) in oxygen-mediated cellular injury and oxidative DNA damage in human pulmonary cells. Oxid. Med. Cell. Longev..

[35] U.S. Srinivas (2019). B.W.Q. Tan, B.A. Vellayappan, A.D. Jeyasekharan, ROS and the DNA damage response in cancer. Redox Biol..

[36] Glorieux C., Buc Calderon P. (2019). Cancer cell sensitivity to redox-cycling quinones is influenced by NAD(P)H: quinone oxidoreductase 1 polymorphism. Antioxidants.

[37] Shibata T., Kokubu A., Saito S., Narisawa-Saito M., Sasaki H., Aoyagi K., Yoshimatsu Y., Tachimori Y., Kushima R., Kiyono T., Yamamoto M. (2011). NRF2 mutation confers malignant potential and resistance to chemoradiation therapy in advanced esophageal squamous cancer. Neoplasia.

[38] Tsuchida K., Tsujita T., Hayashi M., Ojima A., Keleku-Lukwete N., Katsuoka F., Otsuki A., Kikuchi H., Oshima Y., Suzuki M., Yamamoto M. (2017). Halofuginone enhances the chemo-sensitivity of cancer cells by suppressing NRF2 accumulation. Free Radic. Biol. Med..

[39] Zhu J., Wang H., Chen F., Fu J., Xu Y., Hou Y., Kou H.H., Zhai C., Nelson M.B., Zhang Q., Andersen M.E., Pi J. (2016). An overview of chemical inhibitors of the Nrf2-ARE signaling pathway and their potential applications in cancer therapy. Free Radic. Biol. Med..

[40] Baird L., Llères D., Swift S., Dinkova-Kostova A.T. (2013). Regulatory flexibility in the Nrf2-mediated stress response is conferred by conformational cycling of the Keap1-Nrf2 protein complex. Proc. Natl. Acad. Sci. U. S. A..

[41] Ma J., Cai H., Wu T., Sobhian B., Huo Y., Alcivar A., Mehta M., Cheung K.L., Ganesan S., Kong A.N., Zhang D.D., Xia B. (2012). PALB2 interacts with KEAP1 to promote NRF2 nuclear accumulation and function. Mol. Cell Biol..

[42] Wang Q., Ma J., Lu Y., Zhang S., Huang J., Chen J., Bei J.X., Yang K., Wu G., Huang K., Chen J., Xu S. (2017). CDK20 interacts with KEAP1 to activate NRF2 and promotes radiochemoresistance in lung cancer cells. Oncogene.

[43] Zhou Y., Chen Y., Shi Y., Wu L., Tan Y., Li T., Chen Y., Xia J., Hu R. (2023). FAM117B promotes gastric cancer growth and drug resistance by targeting the KEAP1/NRF2 signaling pathway. J. Clin. Invest..

[44] Camp N.D., James R.G., Dawson D.W., Yan F., Davison J.M., Houck S.A., Tang X., Zheng N., Major M.B., Moon R.T. (2012). Wilms tumor gene on X chromosome (WTX) inhibits degradation of NRF2 protein through competitive binding to KEAP1 protein. J. Biol. Chem..

[45] Wang J., Lu Q., Cai J., Wang Y., Lai X., Qiu Y., Huang Y., Ke Q., Zhang Y., Guan Y., Wu H., Wang Y., Liu X., Shi Y., Zhang K., Wang M., Peng Xiang A. (2019). Nestin regulates cellular redox homeostasis in lung cancer through the Keap1-Nrf2 feedback loop. Nat. Commun..

[46] Ge W., Zhao K., Wang X., Li H., Yu M., He M., Xue X., Zhu Y., Zhang C., Cheng Y., Jiang S., Hu Y. (2017). iASPP is an antioxidative factor and drives cancer growth and drug resistance by competing with Nrf2 for Keap1 binding. Cancer Cell.

[47] Tian L., Lu Y., Yang T., Deng Z., Xu L., Yao W., Ma C., Li X., Zhang J., Liu Y., Wang J. (2019). aPKCι promotes gallbladder cancer tumorigenesis and gemcitabine resistance by competing with Nrf2 for binding to Keap1. Redox Biol..

[48] Komatsu M., Kurokawa H., Waguri S., Taguchi K., Kobayashi A., Ichimura Y., Sou Y.S., Ueno I., Sakamoto A., Tong K.I., Kim M., Nishito Y., Iemura S., Natsume T., Ueno T., Kominami E., Motohashi H., Tanaka K., Yamamoto M. (2010). The selective autophagy substrate p62 activates the stress responsive transcription factor Nrf2 through inactivation of Keap1,. Nat. Cell Biol..

