# Supplementary material for: DPP9 regulates NQO1 and ROS to promote resistance to chemotherapy in liver cancer cells
Source: Redox Biol. 2024 Jul 29;75:103292. doi: 10.1016/j.redox.2024.103292 (PMC11345690; doi:10.1016/j.redox.2024.103292)
Supplement: Multimedia component 1 [file mmc1.pdf]

DPP9 regulates NQO1 and ROS to promote resistance to chemotherapy in liver cancer cells

Yunjiang Zhou,[1, #] Yaxin Chen,[1, #] Chenyuan Xuan,[1] Xingyan Li,[1] Yingying Tan,[1] Mengdi Yang,[1] Mengran Cao,[1] Chi Chen,[1] Xing Huang,[2, *] and Rong Hu[1, *]

[1]State Key Laboratory of Natural Medicines, School of Basic Medicine and Clinical Pharmacy, China Pharmaceutical University, Nanjing 210009, China

[2]Department of Pathology, Jiangsu Cancer Hospital & Jiangsu Institute of Cancer Research & Nanjing Medical University Affiliated Cancer Hospital, Nanjing 210009, China

[#]**Authorship note:** Yunjiang Zhou and Yaxin Chen contributed equally to this work.

***Corresponding Author:**

Rong Hu, State Key Laboratory of Natural Medicines, School of Basic Medicine and Clinical Pharmacy, China Pharmaceutical University, Nanjing 210009, China. Email: ronghu@cpu.edu.cn.

Xing Huang, Department of Pathology, Jiangsu Cancer Hospital & Jiangsu Institute of Cancer Research & Nanjing Medical University Affiliated Cancer Hospital, Nanjing 210009, China. Email: polofly2012@njmu.edu.cn.

Conflict of interest

The authors declare no potential conflicts of interest.

Supplemental methods

Western blot assay. Cells and tissues were lysed in RIPA lysis buffer with 1mM PMSF for 1 h on ice and centrifuged at 13,000 rpm for 30 min at 4 °C. The concentrations of total protein were measured by using BCA protein assay kit. Equal amounts of protein were separated by SDS-PAGE and transferred onto polyvinylidene difluoride (PVDF) membranes. Membranes were blocked with 3% BSA in PBS at 37 °C for 2 h and incubated with indicated antibodies overnight at 4 °C. Membranes were then washed three times with PBST followed by incubation with HRP-conjugated secondary antibodies for 1 h at 37 °C. The signals were analyzed using the ECL chemiluminescence detection system (Tanon, Shanghai, China) and Image J software was used to quantify the intensity of the bands. The experiment was independently replicated three times.

MTT assay. To determine the cytotoxicity of chemotherapeutic drugs, cells transfected with the indicated shRNA or overexpression plasmids were plated into 96-well plates at approximately 5,000 cells/well in 100 μL medium, then treated with various concentrations of chemotherapeutic drugs for 48 h. Cell viability was assessed with the MTT assay following the manufacturer's protocol. Cell survival ratio was calculated using Atreated/Acontrol × 100%, where Atreated and Acontrol were the absorbance from treated and control cells after 48 h incubation, respectively. The IC_{50} was taken as the concentration that caused 50% inhibition of cell proliferation and was calculated by SPSS software. The experiment was independently replicated three times.

Plasmids transfection and shRNA interference. For plasmids transfection, cells were seeded in a 6-well plate and maintained in medium without antibiotics. After 24 h, the plasmids and Lipo6000 Transfection Reagent were diluted and mixed in serum-free medium according to the manufacturer's instruction. Then, the plasmids-transfection reagent complexes were added into wells. The medium was replaced after 4 h incubation and the cells were collected to perform western blot analysis after 36 h culture. For shRNA interference, DPP9, NQO1, NRF2, and KEAP1 shRNAs lentiviral particles were diluted in OptiMEM containing 6 μg/ml polybrene, and then were added to cells. After 3 days, 5 μg/ml of puromycin were used to select transfected cells. Cells

transfected with the shRNAs lentiviral particles were seeded into six-well plates and western blot analysis were used to detect the protein levels of DPP9, NQO1, NRF2, and KEAP1.

RNA-sequencing. RNA-sequencing was accomplished in Personalbio. Company (Shanghai, China). In brief, total RNA of tumor tissues were extracted by Trizol Reagent (Invitrogen Life Technologies), the quality of which was measured by NanoDrop spectrophotometer (Thermo Scientific, U.S.A.). To generate sequencing libraries, the mRNA was purified by poly-T oligo-attached magnetic beads from total RNA, which was used to synthesize second strand cDNA. Then, DNA fragments were enriched by PCR reaction that were purified and quantified with Bioanalyzer 2100 system (Agilent Technologies, U.S.A.). Finally, using NovaSeq 6000 platform (Illumina, U.S.A.) to sequenced the constructed sequencing library. RNA-sequencing data generated in this study have been deposited in the Sequence Read Archive (SRA) under the accession number PRJNA1019915.

RNA extraction and qPCR assays. Total RNA was isolated using the RNA isolater Total RNA Extraction Reagent according to the manufacturer's protocol. The concentration and purity of the extracted RNA were measured with the optical densities at 260 and 280 nm. RNA samples were reverse transcribed to cDNA and subjected to quantitative PCR, which was performed with the Light-Cycler_96 RealTime PCR System (Roche) using AceQ qPCR SYBR Green Master Mix. The primer sequences used in this study were shown in Supplemental Table 2. The experiment was independently replicated three times.

CHX-chase assay. Degradation half-life of NQO1 and NRF2 was analyzed by CHX-chase assay. Briefly, cells transfected with the indicated shRNA or overexpression plasmids were incubated with 25 μM of cycloheximide (CHX) to inhibit protein synthesis. Total cell lysates were collected at different times after following treatment with CHX and subjected to western blot analysis with indicated antibodies. The intensity of the bands were quantified by using Image J software. The experiment was independently replicated three times.

Co-immunoprecipitation assay. Co-immunoprecipitation assay was performed using

a standard protocol. Briefly, the cell lysates were incubated with indicated antibodies overnight at 4°C, and then incubated with protein A+G agarose beads for another 4 h at 4°C. Immunoprecipitated proteins were analyzed by western blot with indicated antibodies. The experiment was independently repeated three times.

GST pull-down assay. For the GST pull-down assay, the glutathione sepharose beads conjugated with 500 ng GST or GST-DPP9-WT protein were incubated with 200 ng purified KEAP1 in GST-binding buffer at 4°C for 24 h, followed by washing 3 times for 10 minutes each with GST-washing buffer at 4°C. The samples were separated by SDS-PAGE and stained with coomassie blue. The experiment was independently replicated three times.

Measurement of intracellular ROS level. Intracellular ROS level was detected by using ROS assay kit according to the manufacturer's instructions. The fluorescence intensity was measured by using microplate reader at Ex./Em. = 488/525 nm.

Animal experiments. Female BALB/c nude mice (6 weeks old) were purchased from Beijing Vital River Laboratory Animal Technology Co., Ltd (Beijing, China). All protocols for mice were approved by the Animal Ethics Committee of China Pharmaceutical University (Ethic approval number: 2022-10-019). Cells stably transfected with the indicated shRNA or overexpression plasmids were injected into subdermal space of mice (1×10^7 cells/per mouse). Once the tumors reached 80-100 mm^3, the mice were treated with PBS or Cisplatin (3 mg/kg, 7 times, intraperitoneally) for 28 days. Tumor volume of mice were measured every four days. Tumor volume = (a × a × b)/2 (a, the smallest diameter; b, the largest diameter). For therapeutic experiment, cells were injected into subdermal space of mice (1×10^7 cells/per mouse). Once the tumors reached 80-100 mm^3, mice were randomly allocated into four groups, and then treated with vehicle, cisplatin (3 mg/kg, 7 times, intraperitoneally), dicoumarol (50 mg/kg, 13 times, intraperitoneally) and two drugs for 28 days. Tumor volume of mice were measured every four days. Tumor volume = (a × a × b)/2 (a, the smallest diameter; b, the largest diameter).

Immunohistochemistry assay. Immunohistochemistry (IHC) staining was performed by using immunohistochemistry kit according to the manufacturer’s protocol. Briefly,

5 μm-thick paraffin sections were deparaffinized, rehydrated and washed 3 times with PBS. Antigen repair solution was used to repair the antigen of tissues. Then, tissue sections were treated with 3% hydrogen peroxide at 37°C for 25 min, blocked with 3% BSA at 37°C for 1 h, and incubated with primary antibodies at 4°C overnight. HRP-conjugated secondary anti-rabbit or HRP-conjugated secondary anti-mouse antibody was added and incubated at room temperature for 1 h. The tissue sections were stained with DAB substrate and counterstained with hematoxylin. All images were acquired by an inverted microscope (Nikon, Japan). The experiment was independently replicated three times.

Immunofluorescence. For cellular immunofluorescence assay, cells were fixed in 4% paraformaldehyde for 20 min, and washed with PBS for 3 times. Then, cells were incubated with Triton X-100 for 5 min, blocked with 3% BSA at 37°C for 1 h, and incubated with primary antibodies at 4°C overnight. The cells were washed with PBS for 3 times and incubated with keyFluor 488 Goat Anti-Mouse and Cy3 conjugated Goat Anti-Rabbit IgG antibodies for 1 h. Next, the cells were washed with PBS for 3 times and incubated with DAPI. All images were acquired by an inverted microscope (Nikon, Japan). The experiment was independently replicated three times.

Clinical samples. A total of 108 liver cancer clinical samples were collected from patients after receipt of written informed consent in accordance with a protocol approved by Ethics Committee of Nanjing Medical University Affiliated Cancer Hospital. IHC staining was used to analyze the protein levels of DPP9 and NQO1 in tumor tissues and matched adjacent normal tissues. Each specimen was assigned a score according to the intensity of the staining (no staining = 0, weak staining = 1, moderate staining = 2, strong staining = 3) and the extent of stained cells (0% = 0, 1-24% = 1, 25-49% = 2, 50-74% = 3, 75-100% = 4). When the stain was homogenous, the IHC score was determined by multiplying the intensity score with the extent score of stained cells. When the stain was heterogeneous, we scored it as follows: each component was scored independently and summed for the results. A score of 6 or less was defined as a low IHC score, and a score higher than 6 was defined as a high IHC score. All of staining was assessed by pathologist blinded to the origination of the samples and

subject outcome.

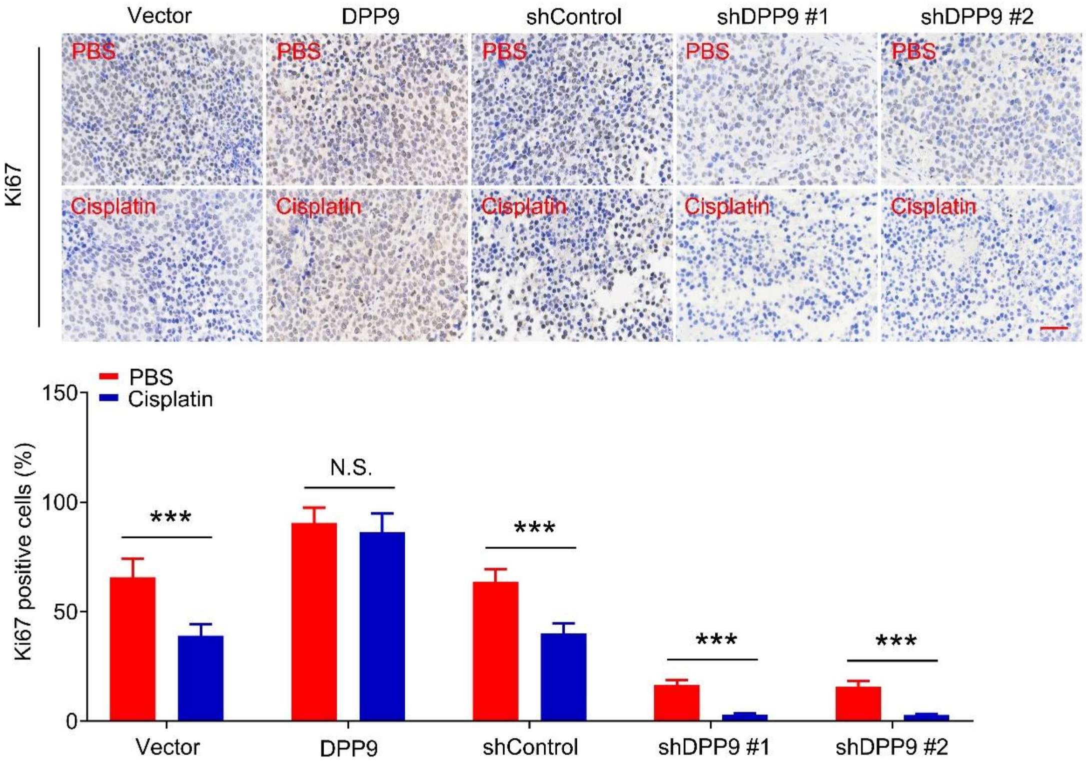

Supplemental Fig. 1. The positive rates of Ki67 in SK-Hep-1 cell-derived xenograft tissues of Vector + PBS, Vector + cisplatin, DPP9 + PBS, DPP9 + cisplatin, shControl + PBS, shControl + cisplatin, shDPP9 #1 + PBS, shDPP9 #1 + cisplatin, shDPP9 #2 + PBS, and shDPP9 #2 + cisplatin groups. Scale bar = 50 µm. Data are shown as mean ± SD. ****P* < 0.001 means significant difference. N.S. means no significant difference.

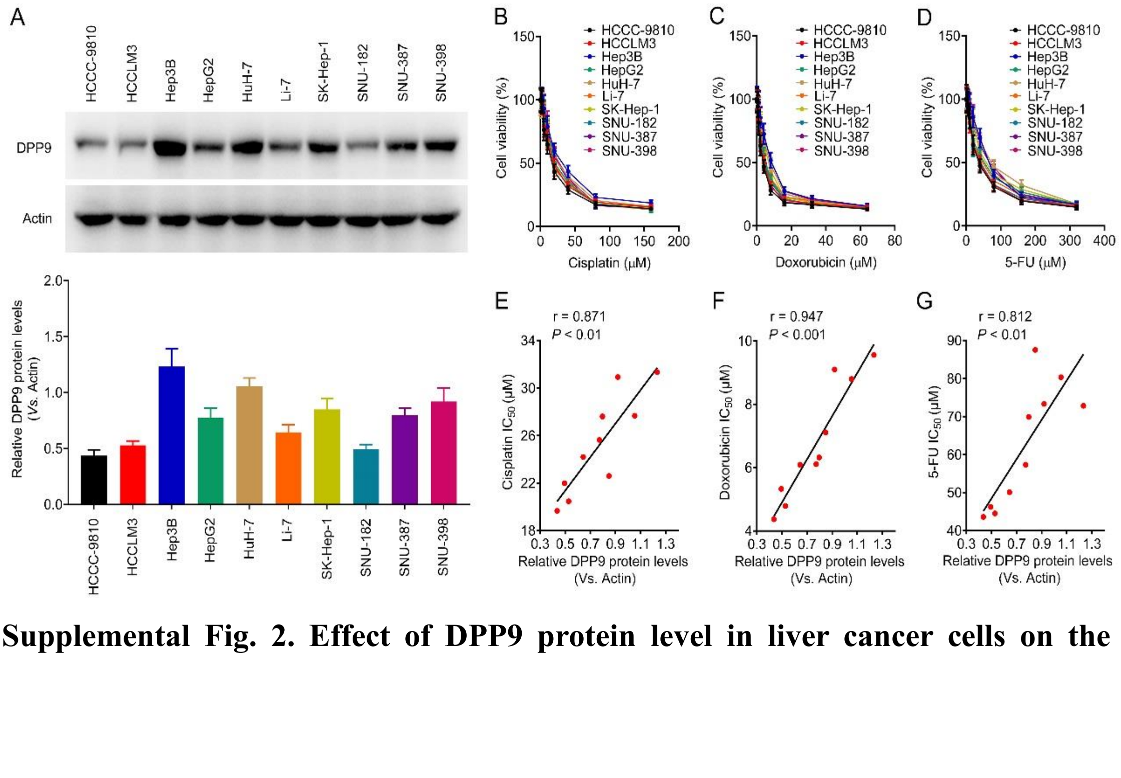

Supplemental Fig. 2. Effect of DPP9 protein level in liver cancer cells on the

efficacy of chemotherapy drugs. (A) DPP9 protein level in liver cancer cells. (B-D) Efficacy of chemotherapy drugs (cisplatin, doxorubicin, and 5-FU) in liver cancer cells. (E-G) Correlation between DPP9 protein level and IC_{50} of chemotherapy drugs in liver cancer cells. Data are shown as mean ± SD. $P < 0.01$ or $P < 0.001$ means significant difference.

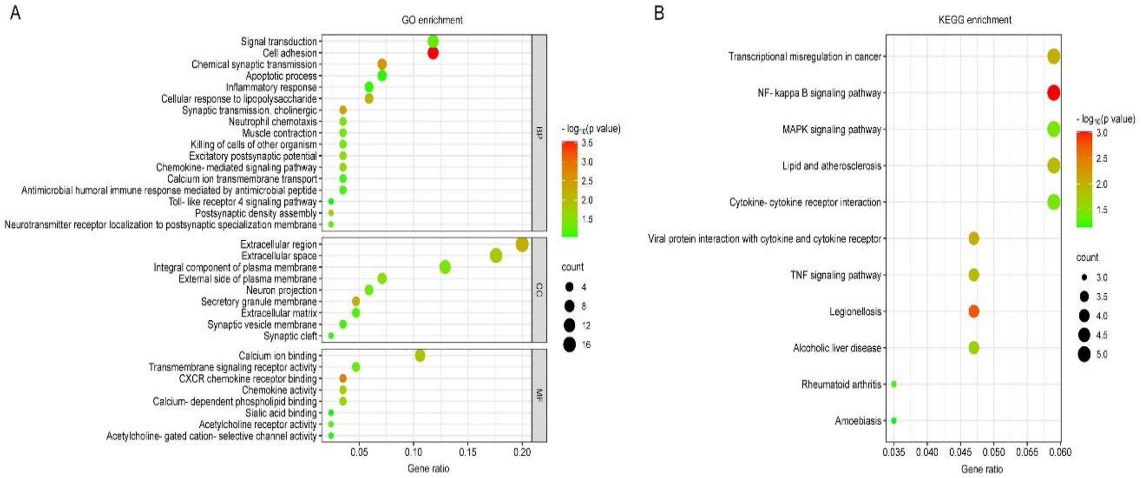

Supplemental Fig. 3. Gene Ontology (GO) and Kyoto Encyclopedia of Genes and Genomes (KEGG) enrichment analyses for the genes with significant changes identified by transcriptomic sequencing analysis. (A) GO enrichment analysis. (B) KEGG enrichment analysis.

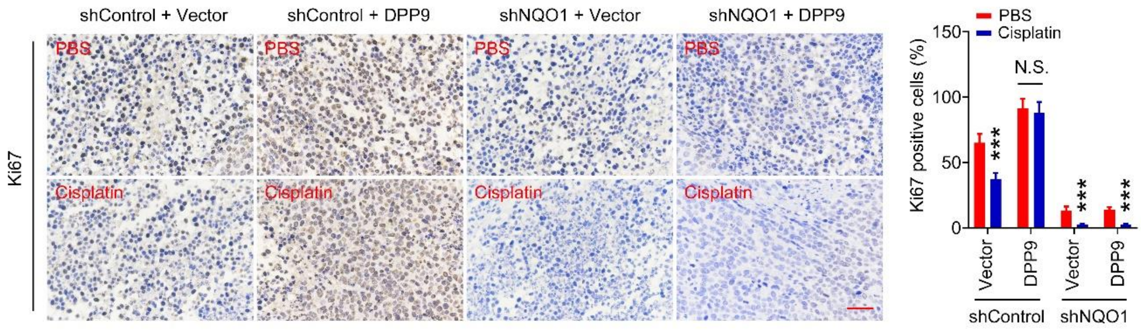

Supplemental Fig. 4. The positive rates of Ki67 in SK-Hep-1 cell-derived xenograft tissues of shControl + Vector + PBS, shControl + Vector + cisplatin, shControl + DPP9 + PBS, shControl + DPP9 + cisplatin, shNQO1 + Vector + PBS, shNQO1 + Vector + cisplatin, shNQO1 + DPP9 + PBS, and shNQO1 + DPP9 + cisplatin groups. Scale bar = 50 μm. Data are shown as mean ± SD. ***$P < 0.001$ means significant difference *vs.* PBS group. N.S. means no significant difference.

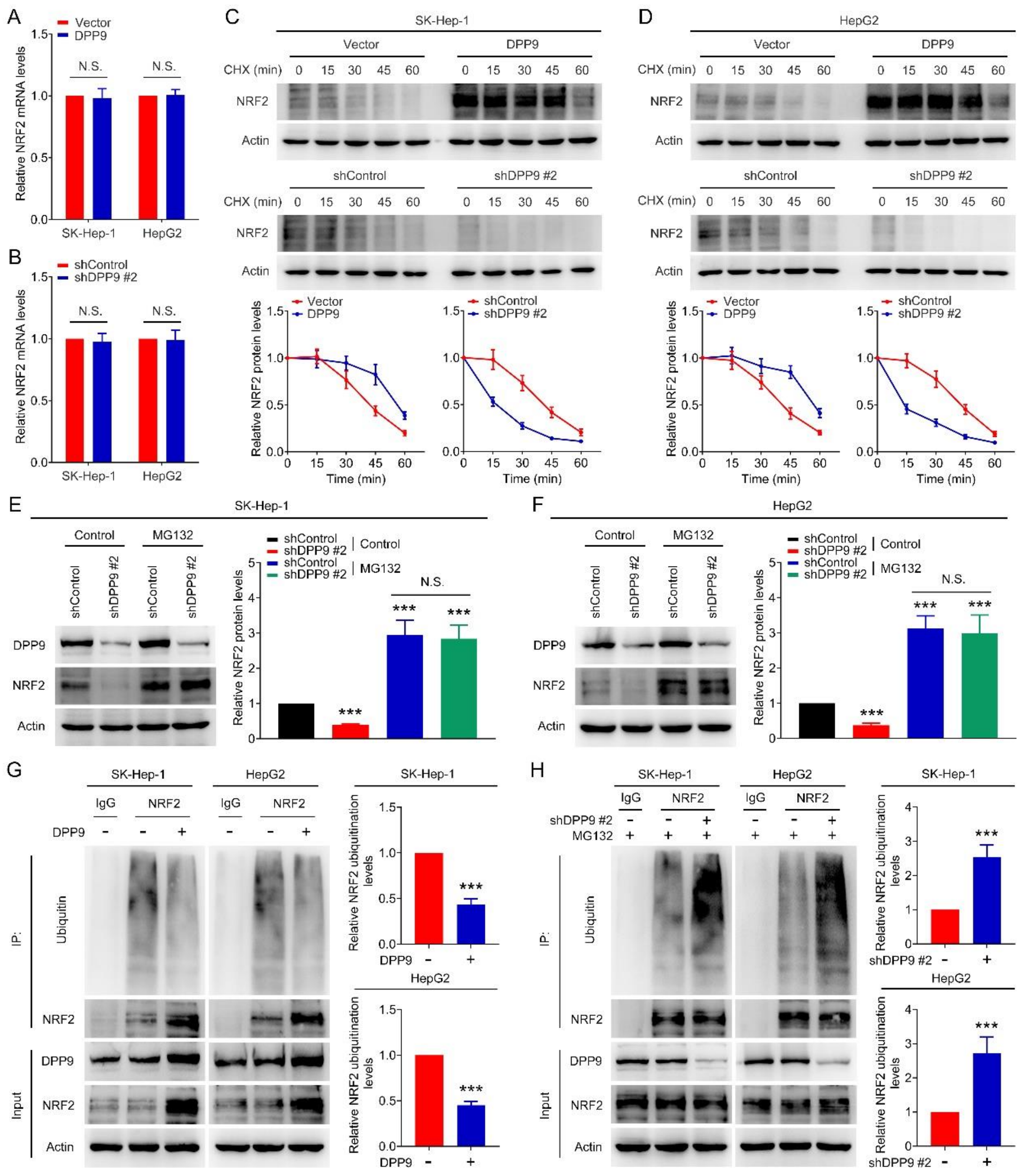

Supplemental Fig. 5. DPP9 up-regulates NRF2 protein levels in liver cancer cells by inhibiting NRF2 ubiquitination degradation. (A-B) NRF2 mRNA levels in SK-Hep-1 and HepG2 cells with DPP9 overexpression and silencing. (C-D) NRF2 protein degradation rate in SK-Hep-1 and HepG2 cells with DPP9 overexpression and silencing. (E-F) DPP9-silenced SK-Hep-1 and HepG2 cells were treated with or without MG132 (20 μM), NRF2 protein levels in cells were detected by western blot. (G-H) NRF2 ubiquitination levels of SK-Hep-1 and HepG2 cells with DPP9 overexpression and silencing. Data are shown as mean ± SD. ***P < 0.001 means significant difference *vs.* Vector or shControl. N.S. means no significant difference.

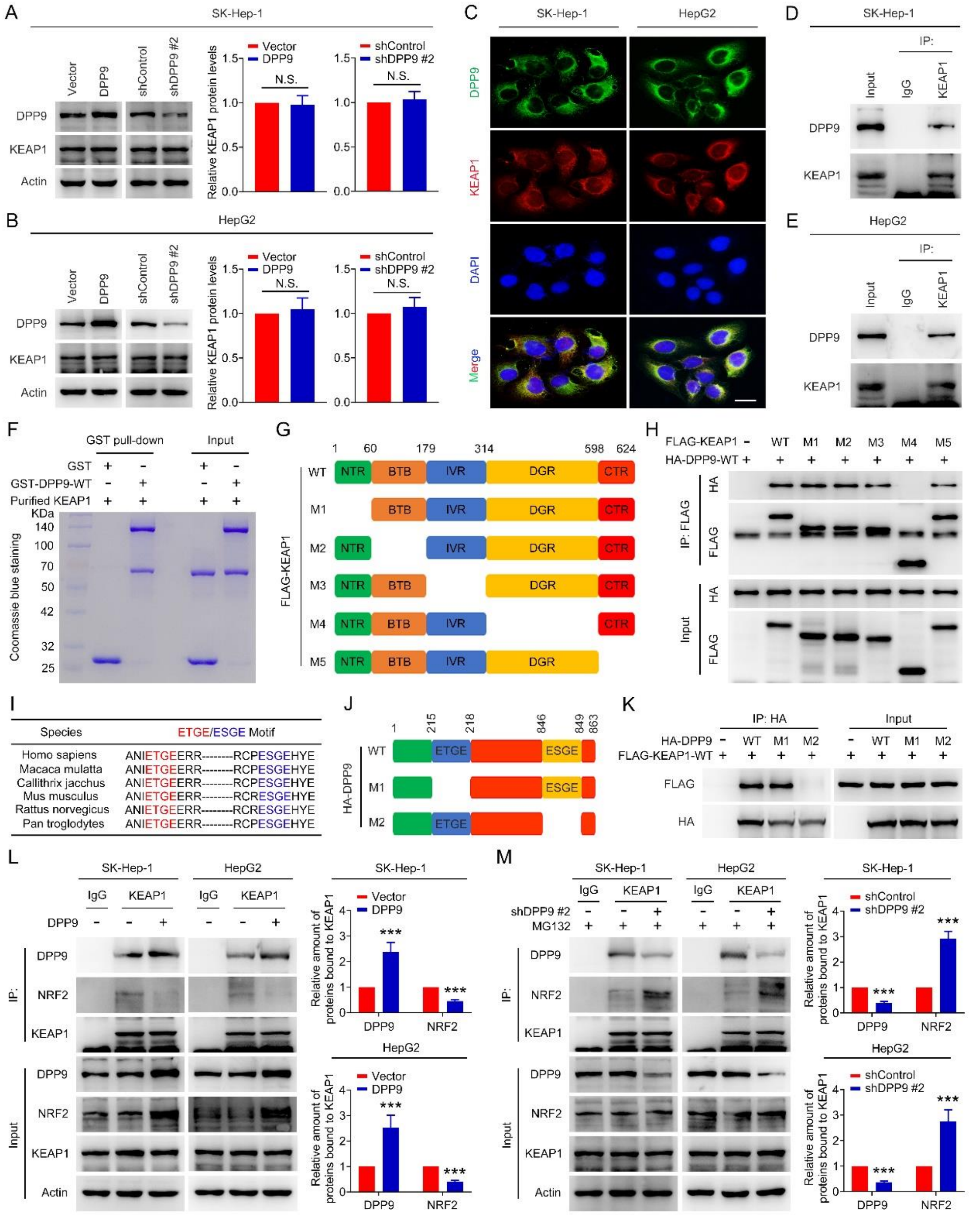

Supplemental Fig. 6. DPP9 competes with NRF2 to bind KEAP1 in liver cancer cells. (A-B) KEAP1 protein levels in SK-Hep-1 and HepG2 cells with DPP9 overexpression and silencing. (C) Localization of DPP9 and KEAP1 in cells. Scale bars = 10 μm. (D-E) Binding of endogenous DPP9 to KEAP1 in SK-Hep-1 and HepG2 cells. (F) Binding of purified GST-DPP9-WT to purified KEAP1. (G) Diagram of WT and truncated mutants of KEAP1. (H) Binding of HA-DPP9-WT to FLAG-KEAP1-WT or truncated mutants in HEK293T cells. (I) ETGE and ESGE motifs of DPP9 in different species. (J) Diagram of WT and truncated mutants of DPP9. (K) Binding of FLAG-KEAP1-WT to HA-DPP9-WT or truncated mutants in HEK293T cells. (L-M) Binding levels of DPP9 or NRF2 to KEAP1 in SK-Hep-1 and HepG2 cells with DPP9

overexpression and silencing. Data are shown as mean ± SD. ***$P < 0.001$ means significant difference *vs.* Vector or shControl group. N.S. means no significant difference.

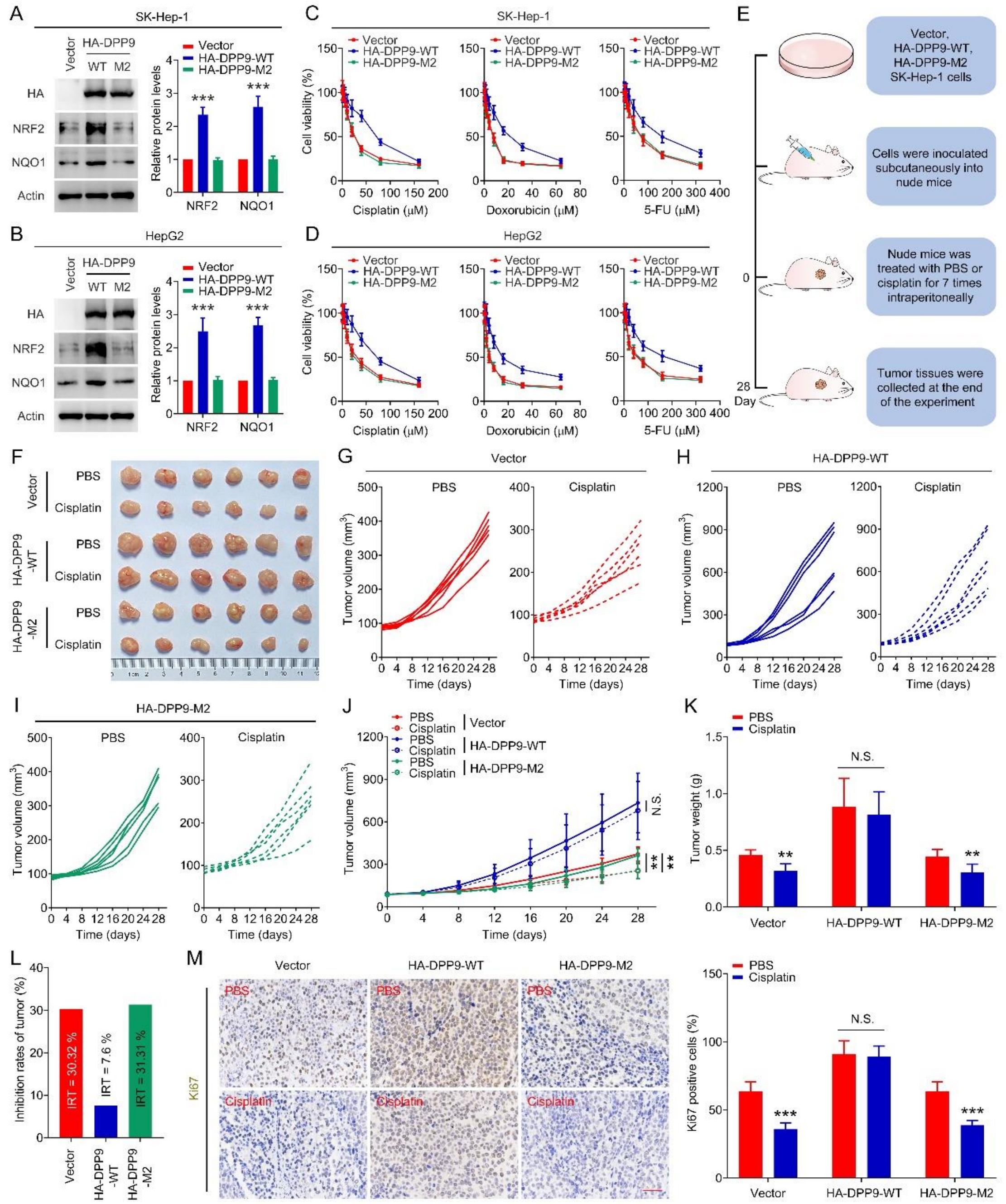

Supplemental Fig. 7. The ESGE motif of DPP9 up-regulates NRF2 and NQO1 protein levels and weakens the responses of cells to chemotherapy. (A-B) NRF2 and NQO1 protein levels in SK-Hep-1 and HepG2 cells transfected with HA-DPP9 WT or ESGE-truncated mutant plasmids. (C-D) Toxic effects of chemotherapy drugs (cisplatin, doxorubicin, and 5-FU) on liver cancer cells transfected with HA-DPP9 WT or ESGE-truncated mutant plasmids. (E) Schematic diagram of the in vivo study. (F) Image of tumors. (G-J) Growth curves of tumor volume. (K) Tumor weight. (L) Inhibition rates

of tumor. (M) Immunohistochemistry staining. Scale bar = 50 μm. Data are shown as mean ± SD. **$P < 0.01$ or ***$P < 0.001$ means significant difference *vs.* Vector or PBS group. N.S. means no significant difference.

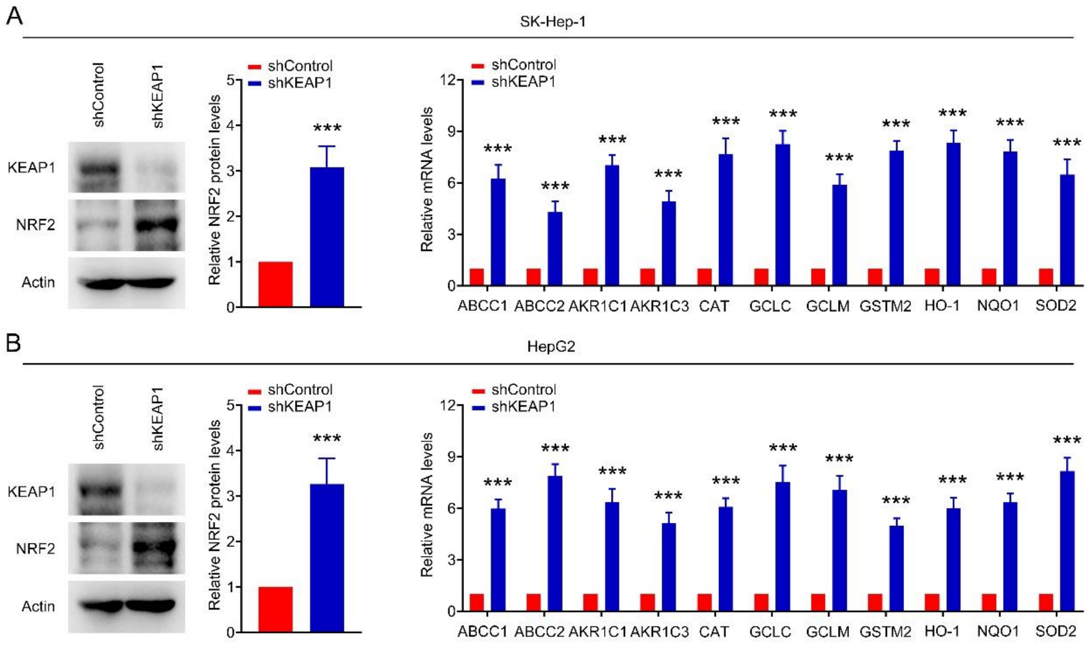

Supplemental Fig. 8. Effect of KEAP1 knockdown on NRF2 downstream target genes in liver cancer cells. (A) The levels of NRF2 downstream target genes in SK-Hep-1 cells with KEAP1 knockdown. (B) The levels of NRF2 downstream target genes in HepG2 cells with KEAP1 knockdown. Data are shown as mean ± SD. ***$P < 0.001$ means significant difference *vs.* shControl group.

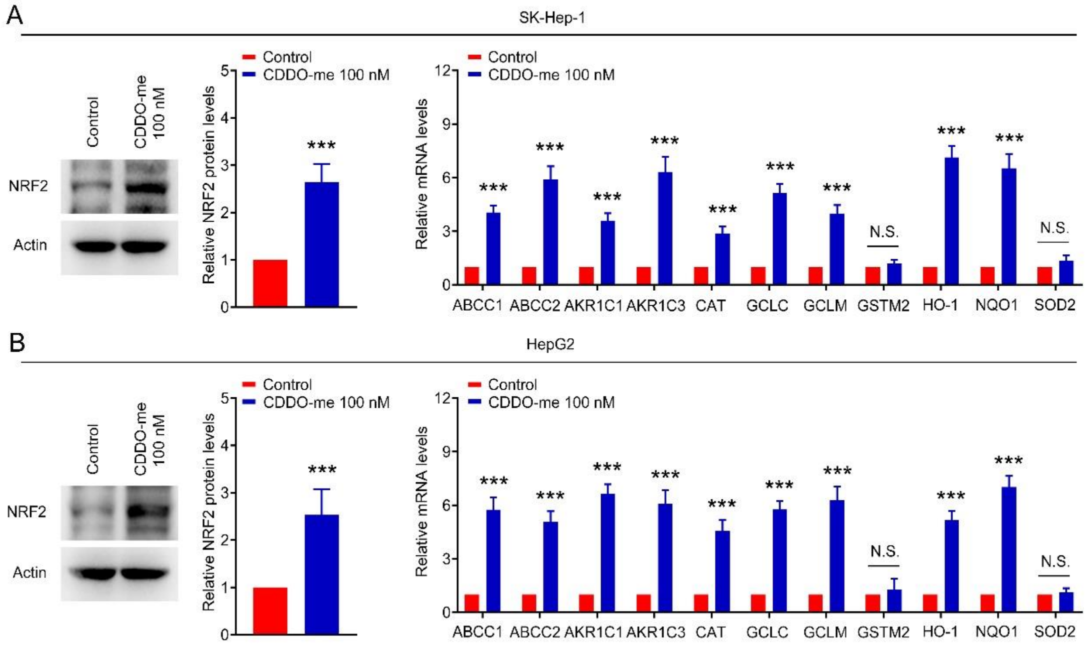

Supplemental Fig. 9. Effect of CDDO-me on NRF2 downstream target genes in liver cancer cells. (A) The levels of NRF2 downstream target genes in SK-Hep-1 cells

treated with 100 nM of CDDO-me. (B) The levels of NRF2 downstream target genes in HepG2 cells treated with 100 nM of CDDO-me. Data are shown as mean ± SD. ****P* < 0.001 means significant difference *vs.* Control group. N.S. means no significant difference.

Supplemental Table 1. Cox regression analysis of risk factors for cancer-related death.

**Variables**	**Univariate analysis**		**Multivariate analysis**	
	**HR (95% CI)**	***P***	**HR (95% CI)**	***P***
**Sex**				
Male	1			
Female	0.385 (0.092, 1.608)	0.191		
**Age**				
< 60 years	1			
≥ 60 years	1.108 (0.559, 2.193)	0.769		
**Tumor size**				
< 6 cm	1		1	
≥ 6 cm	2.774 (1.4, 5.498)	0.003	1.576 (0.564, 4.401)	0.385
**Clinical stage**				
1-2	1		1	
3-4	3.161 (1.504, 6.646)	0.002	0.139 (0.011, 1.748)	0.127
**T status**				
T1-T2	1		1	
T3-T4	3.088 (1.496, 6.374)	0.002	6.637 (0.498, 88.513)	0.152
**N status**				
N0-N1	1			
N2-N3	1.278 (0.493, 3.31)	0.614		
**M status**				
M0	1		1	
M1	9.675 (2.859, 32.733)	< 0.001	17.958 (3.418, 94.353)	0.001

**Protein IHC score**				
DPP9^{low}/NQO1^{low}	1		1	
DPP9^{low}/NQO1^{high}	2.734 (0.248, 30.15)	0.412	2.52 (0.226, 28.059)	0.452
DPP9^{high}/NQO1^{low}	6.176 (0.642, 59.394)	0.115	5.238 (0.523, 52.485)	0.159
DPP9^{high}/NQO1^{high}	13.651 (1.853, 100.564)	0.01	11.142 (1.37, 90.635)	0.024

Supplemental Table 2. The sequences of primers used in this study.

Gene	Forward (5′-3′)	Reverse (5′-3′)
ABCC1	CTCTATCTCTCCCGACATGACC	AGCAGACGATCCACAGCAAAA
ABCC2	CTTGGGCTTCCTATGGCTCC	ATCGAACAGCAGGGACTGTG
AKR1C1	TTCATGCCTGTCCTGGGATTT	CTGGCTTTACAGACACTGGAAAA
AKR1C3	GGATTTGGCACCTATGCACCTC	CTATATGGCGGAACCCAGCTTCTA
ANXA10	TTGTGGAGACTATGTGCAAGGA	GGTATGCCTCTGCAATCATCAT
CAT	CGGAGATTCAACACTGCCAATG	TTCTTGACCGCTTTCTTCTGGA
GCLC	GGACAAGAATACACCATCTCCA	ATACTGCAGGCTTGGAATGTC
GCLM	GGGAACCTGCTGAACTGG	CTGGGTTGATTTGGGAACTC
GSTM2	ACA ACCTGTGCGGGGAATC	AGCTTCAGCATTTCAGGGAGTG
HO-1	CTTTCAGAAGGGCCAGGTGA	GTAGACAGGGGCGAAGACTG
HSPA6	CAAGGTGCGCGTATGCTAC	GCTCATTGATGATCCGCAACAC
NQO1	GGTTTGGAGTCCCTGCCATT	TTGCAGAGAGTACATGGAGCC
NRF2	CAGCTTTTGGCGCAGACATT	GACTGGGCTCTCGATGTGAC
SOD2	TGGGGTTGGCTTGGTTTCAA	GGAATAAGGCCTGTTGTTCCTTG
TLL2	TTGAAGAGCAGGCATCTGAGA	CGTAGGGGATGACTCCTCCAG
